# A GIS based approach to long bone breakage patterns derived from marrow extraction

**DOI:** 10.1371/journal.pone.0216733

**Published:** 2019-05-31

**Authors:** T. Stavrova, A. Borel, C. Daujeard, D. Vettese

**Affiliations:** Histoire Naturelle de l’Homme Préhistorique (HNHP, UMR 7194), Sorbonne Universités, Muséum national d’Histoire naturelle (MNHN), Homme et Environnement, CNRS, Institut de Paléontologie Humaine, Paris, France; Max Planck Institute for the Science of Human History, GERMANY

## Abstract

In archaeological assemblages the presence of percussion marks, on the surface of long bones, is an indicator of long bone marrow extraction. The form, quantity and distribution of percussion marks are analysed to gain a better understanding of the marrow extraction process. Patterns of bone percussion damage in archaeological assemblages may highlight standardized actions, possibly related to butchery traditions. However, additional factors could underlie these patterns and should also be considered. In this article we test intuitiveness as a factor in appearance of percussion mark patterns, to see if patterns can appear when bones are being fractured without prior experience with bone fracture properties. To test this hypothesis, for this study we selected a sample of 40 cattle (*Bos taurus)* long limb bones from a large bone breakage experiment (400 long limb bones), where participants had no previous experience in bone breakage and may thus have broken bones intuitively. We used Geographic Information System (GIS) software to analyse the distribution of percussion marks. Using ArcGIS Spatial Analysts tools, we identified and quantified significant concentrations of percussion marks. Results show that percussion mark patterns emerge for the same bone element, and that specific sides and zones were recurrently selected by experimenters. The distribution of patterns varies among the different long bone elements, and we attribute this variance to an adjustment to bone morphology. In addition, we calculated and identified bone damage patterns resulting from hammerstone percussion. Crossing bone survivorship with percussion mark patterns enabled us to recognise and evaluate the effects of fragmentation and surface visibility in controlled experimental conditions. The GIS method facilitates comparisons between different variables and provides a sophisticated visual representation of results. Enlarging the sample will allow to constitute a more substantial analogous model for fossil assemblages.

## Introduction

Over the past decades, archaeology and human behaviour studies have progressively incorporated research tools and analytical approaches from geospatial sciences. More than 30 years ago, the term GIS (Geographic Information System) was introduced to archaeology [1], and its application to the field has received mixed reviews over the years. Numerous articles discuss its effectiveness and advantages but also the uncertainty of the system [2–5]. However, since the late 2000s, GIS has rapidly gained momentum and is now an important asset to the archaeologist’s tool set.

A GIS is a computer system capable of assembling, storing and manipulating, analysing and displaying geographically referenced information, i.e. data identified according to their location [6]. As stressed by many other definitions [7–9], the key word in GIS is geography and working with this system requires the terrestrial projection of data, meaning a system of coordinates in space that can correspond to latitudes and longitudes. Still, researchers from different fields grasped the potential of the software for non-geographical data and recent studies have used the system in an alternative way to apply the analytical capabilities of GIS to their subjects [10–12]. In a similar manner, we used GIS to illustrate and analyse the distribution of percussion marks produced during bone marrow extraction. The application of GIS to bone was introduced to archaeology by Marean and colleagues [13] to calculate the MNE based on anatomically overlapping specimens. All specimens are digitally drawn in vector mode over templates and then converted into pixels. The maximum number of pixel overlaps detected by the software indicates the MNE. The digitization of the method automated the process of MNE counts and made it appropriate for large assemblages which are otherwise difficult and time consuming to study. This innovative approach received a lot of attention. Lyman [14] argued that MNE calculation with the software could be easily biased by a lack of precision during the drawing of specimens. Others found it valid and continued to develop and adapt the method to more recent versions of GIS [15] and expand its applicability to the analysis of cut marks [16–17].

More recently, Parkinson and colleagues [18–20] proved the software to be well suited to documenting gross bone damage and tooth mark distribution in carnivore-modified assemblages. Spatial point pattern analysis, within GIS, indicates the distribution of tooth marks with greater resolution than previous methods. This allows comparisons with bone portion data. Parkinson’s work complements a series of studies that have been developing methodologies for the past three decades to differentiate between human and non-human carnivore modifying agency [21–27]. Her experimental work analysing carnivore marks with GIS provides analogous models for the interpretation of zooarchaelogical assemblages and helps to identify the modifier or modifiers and their order of access. In recent years, many archaeological and paleoanthropological research teams have conducted bone breakage experiments subsequent to recurrent comments that bone fracturing and its diagnostic features (pits, notches, adhering flakes and crushing marks) are underexplored. Experiments and consequently developed methodologies broaden the possibilities and provide venues to explore bone breakage processing intensity and butcher investment [28], distinguish grease rendering from marrow extraction [29], and identify cultural patterns in bone breakage processes [30–34]. Recently, Blasco and colleagues [30] have argued that systemized patterns of long bone breakage for recovering marrow can help to identify butchery traditions in Neanderthal groups. To further the subject, of Neanderthal butchery know-how, Vettese [32] conducted a bone breakage experiment (400 long bones) in 2017 with participants that had no previous experience in bone breakage. The main aim of the experiment was to test whether recurrent percussion mark patterns will appear on the bone surface and if so to what extent, when the experimenters have no preconceived concepts on how to perform the task i.e. they will perform the task based on their intuition.

For this study, we selected a sample from this large experimental assemblage to analyse percussion mark patterns with GIS. We adapted and expanded the methods developed for GIS to a recent and updated version of the ArcGIS software (ArcMap 10.4) to explore and demonstrate some of the analytical possibilities available with this software when applied to percussion marks. More precisely, we set out to:

- Develop an effective GIS protocol to standardize the documentation process (database and visual representation) for fragments and percussion marks deriving from marrow extraction from long limb bones.
- Create an analytical workspace that allows a spatial link between the non-geographic database and its visual representation.
- Explore GIS abilities to record, quantify and illustrate bone fragments and bone survivorship.
- Explore new analytical methods to illustrate, quantify and analyse percussion marks and patterns of percussion marks.
- Test different hypotheses related to intuitive fracturing.
- Choose a model that can provide valuable insights for analogous archaeological assemblages.

Here, we do not examine the issue of how to diagnose or define percussion marks. Rather, our goal is to address the issue of how to record and then analyse them using GIS and ultimately quantify the sample and concentrations of percussion marks for meaningful behavioural analysis, through comparisons with other modern control assemblages or archaeological assemblages.

## Material and methods

### Material

The studied sample is composed of 40 long limb bones from adult *Bos taurus* species. A series of ten bones was selected from the following elements: humerus, radius-ulna, femur and tibia. Bones were supplied by a slaughterhouse where carcasses were defleshed by a professional butcher. Metapodials are absent from the experiment as they were cut into two parts during the defleshing process, and were thus useless for this experiment.

Non-trained experimenters broke a series of ten long bones to a sufficient extent to remove all marrow (Table 1). Bones were broken with a non-modified quartzite hammerstone (pebble) against a limestone anvil. The periosteum was not removed prior to marrow removal. The experimental bone breakage was performed in a designated area, property of the Muséum National d'Histoire Naturelle, for which all necessary permits were obtained.

**Table 1:** General information regarding volunteers and bone elements.

*Volunteer sex*	*Volunteer age*	*Element*	*Series n°*	*Number of right elements*	*Number of left elements*
*female*	29	Humerus	1	3	7
*male*	40	Radius-ulna	2	6	4
*male*	25	Femur	3	6	4
*male*	30	Tibia	4	4	6

After breakage, all the fragments of each element were collected in a single bag associated with an identification code. This code provides information concerning the series and element numbers, which facilitated later identification and refitting. Broken bones were cleaned by the Service of Osteology and Taxidermy Treatment (SPOT) of the National Museum of Natural History of Paris (MNHN). Each bone was treated separately to avoid the loss of small fragments and dissociation from identification codes. Finally, we labelled each fragment with a permanent marker.

### Methods

#### Template/Image preparation and georeferencing

**Fig 1:**
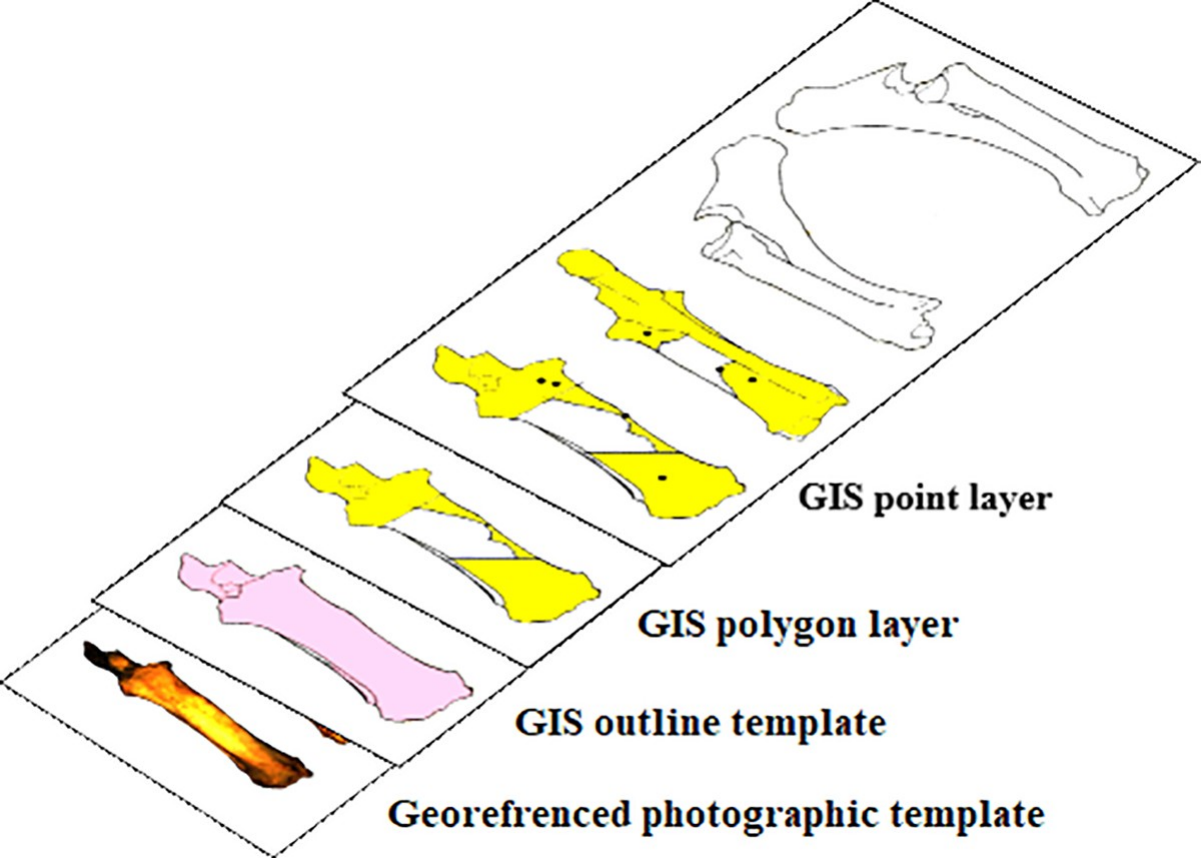
Superposition of GIS layers drawn over the photographic template.

As a base for GIS analysis, we used photographic images representing the four-sided visualization of bone elements [35]. The templates were georeferenced in GIS. As bones are not part of geography, reference coordinates are assigned arbitrarily but they should be specified so that, data can be shared and made compatible with other GIS systems if needed. We used the WGS1984 Greenwich projection. A GIS polygon layer was created over each bone image (Fig 1), replicating the bone silhouette from four views (anterior, lateral, medial and posterior). These templates represent the basis for all further analysis. It is important to note that the area defined by these templates does not reflect the actual bone area. The measurements of all bones from the same element show slight variation and when projected with the software, for the purpose of standardization and superposition of layers, their metric measurements undergo deformations.

#### Bone refitting and analysis

We examined each specimen and percussion mark individually and in relation to each other through systematic bone refitting for every element. Each fragment was measured (mm) and weighed (g), then labelled with its series number, element number and an individual specimen number (e.g., 1-1-1 = series number-element number-fragment number). Bone refitting generates more precise drawing and localization of fragments and percussion marks in GIS. It also enhances the identification and comprehension of percussion marks, as in some cases, a mark can be obliterated by subsequent blows in the same area, which may be required to fracture the bone completely. When refitting two or more fragments, at times we re-evaluated our judgment based on the analysis of a single fragment, i.e., a single notch can be divided and appear on two (or more) fragments. With refitting we count this as a single percussion mark.

We observed all specimens for surface modifications under a strong light (60 W), first with the naked eye; then with a 15-20X lens. Identification and characterization of percussion modifications are based on descriptions and illustrations of bone surface modifications e.g., [23] [28] [31–32], [36–38].

We recorded the following modifications regrouped in this study under the term percussion marks:

- Percussion pits,
- Notches,
- Adhering flakes,
- Crushing marks,
- Cortical conchoidal scars.

Pseudo-notches and peeling were not taken into consideration because they are not direct marks but successive results of fractures. We recorded the marks due to blows and counterblows. We did not observe chop-marks in our sample due to the roundness of the hammerstone pebble and in spite of the anvil’s sharp edge.

#### Drawing fragments in GIS

All identifiable fragments are digitally drawn in GIS, regardless of size. They are drawn in vector mode as polygon features relative to the anatomical landmarks of the previously prepared templates (Fig 1). As defined by previous studies [13], [16], [18–20], identifiable fragments are fragments for which the element and side can be identified and precisely situated on the bone template. We would add to this definition that the outline of the drawn fragment is determined by the outline of the cortical surface of the fragment. This may seem irrelevant, but often fragments that lost part of their cortical surface but conserved medullary surfaces overlap with cortical surface from other fragments from the same bone element, and bias the results. Therefore drawing only the cortical surface circumvents any possible overlapping of fragments of the same bone. Fragments from the same bone element were drawn in a single layer. The radius and ulna were each drawn in a separate layer, in order to eliminate possible errors when calculating bone survivorship or spatial statistics, even though they are fused and generally considered as one element.

An attribute table storing data for every feature, added by the analyst, is linked to every layer. We recorded the reference number for each fragment and the three dimensions (length, width, thickness) were measured for each specimen. Shaft fragments were differentiated by size (Table 2) and circumference classes (Table 3) according to Villa and Mahieu (1991) [38].

**Table 2:** Definition of categories separating shaft fragments by size according to Villa and Mahieu (1991) [38].

L1	Shaft fragments that are less than one-fourth the original length of the complete shaft
L2	Shaft fragment length is comprised between one-fourth and one-half of the complete shaft.
L3	Shaft fragment length is between one-half and three-fourths of the complete shaft
L4	Shaft fragment is more than three-fourths, essentially a complete or almost complete shaft.

**Table 3:** Definition of categories separating shaft fragments by circumference size according to Villa and Mahieu (1991) [38].

C1	Bone circumference is less than half of the original
C2	Bone circumference is more than half, but not a complete circumference, in at least a portion of the bone length
C3	Bone circumference is complete in at least one portion of the bone length

**Drawing percussion marks.** Separate point layers were created to illustrate all the identified percussion marks on the bone surface (Fig 1). We marked them with a point symbol which is also linked to an attribute table with information about the mark:

- n° bone element and n° fragment on which the percussion mark appears,
- Type of percussion mark (notch, pit, adhering flake and crushing),
- Percussion mark maximum length,
- Percussion mark maximum width,
- Percussion mark maximum thickness,
- Notch depth: three different categories: d1 (<½initial cortical height), d2 (>½and<1 initial cortical height) and d3 (= 1, total cortical height),
- Notch form, three categories: fe1 (= symmetric to the fracture, longest depth is in the centre part of the notch when the diaphysis is observed from a vertical angle); fe2 (= asymmetric, longest depth of the notch is on the distal side) and fe3 (= asymmetric, longest depth is on the proximal part),
- Text column for additional observations.

Points illustrating all the percussion marks are placed in the centre of the observed impact recording anatomical landmarks and the drawings of the fragments, e.g., points representing notches are situated in the middle of what is regarded as the fracture edge of the percussion mark. Points representing the rest of the percussion marks are situated in the centre of the observed impact area. We do not consider that the origin of the impact is at the centre of each percussion mark, but this choice was essential for standardizing the drawing process. We chose point features over line and polygon features to standardize and facilitate visualization and to fully exploit GIS spatial pattern analyses.

**Bone survivorship.** The GIS software can calculate the area defined by the outline of a polygon; therefore, we could calculate the area of each drawn fragment. With this information, we adapted the equation from Abe *et al*. [16] to calculate the frequency of cortical bone survivorship for each element:

Sum of fragments surface / whole bone surface *100 = preserved cortical bone surface %.

To illustrate cortical bone survivorship, we follow Marean’s procedure for MNE calculations [13], to which we made modifications available with the more recent versions of ArcMap. Previous studies [13], [15], [18–20] mainly used grid (pixel) overlapping, which requires converting vector to raster files, making the process more time consuming. Our method, detailed in supplementary material, consists of directly overlaying polygons to calculate cortical bone survivorship.

#### Percussion mark frequency

The frequency of percussion marks was calculated in relation to the preserved area for each element of each series:

Numberofpercussionmarks/preservedbone%*100=percussionmark%

This equation is an adaption of the Abe *et al*. [16] equation for calculating cut mark frequency. It was developed to improve frequency calculations by taking into consideration the fragmentation problem. Abe *et al*. [16] state that by dividing the number of cut marks by the preserved surface area, cut mark frequencies would closely match the original frequencies. Thus, “we can correct the number of cutmarks by the amount of examined surface area, much as demographers standardize population size by estimating population density”. In the same way as Lyman [14], we do not agree that this formula can be used to estimate the frequency of marks that have been destroyed, but we consider that it is a fair estimation of the frequency of preserved marks.

#### Patterns and clusters

**Fig 2:**
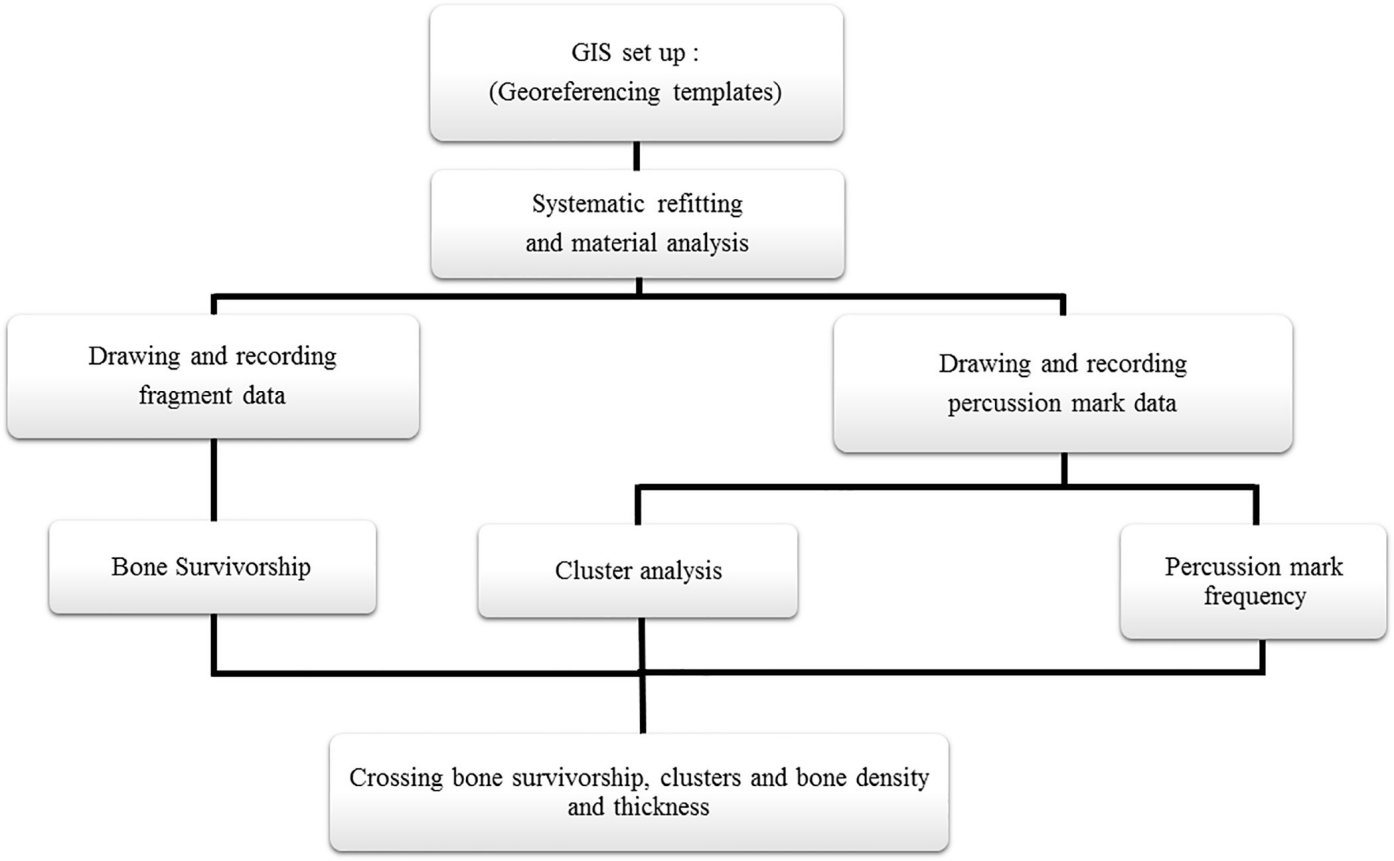
GIS stages for percussion mark analysis.

When confronted with visual spatial data, we inherently organize, group, differentiate and transform these data into information. “Mapping” them like some previous studies [33–34] might result in a sense of the overall pattern of features and their associated values, but GIS spatial tools estimate concentrations of percussion marks by evaluating the interdistance between features. Calculating a statistic with GIS quantifies the pattern, which makes it easier to compare patterns and gain a better understanding of clusters (Fig 2). For this purpose, we used ArcGIS following spatial analyst and statistic tools:

**Analysing patterns with Average Nearest Neighbour Distance (NND).** We used the Average NND tool to test for significant spatial clusters following Parkinson and colleagues’ method [18–20]. The tool calculates a nearest neighbour index based on the average distance from every point (percussion mark) to its nearest neighbouring feature. If the average distance between points is less than the average for a hypothetical random distribution, the distribution is considered to be clustered. If it is greater than one, the pattern is considered to be dispersed. The Z score represented by colour codes (S1 and S2 Tables) determines the value of probability that the observed pattern is significantly different from a random pattern [6]. The obtained values are sensitive to changes to the study area or changes to the Area parameter. The results are most appropriate when the study area is fixed. The defined area in which we tested for clusters is the bone surface calculated with the GIS polygon template. The NND was calculated for each view (anterior, posterior, medial and lateral) separately to accurately represent the surface area of the bone.

We used the NND tool to test for:

- Clusters taking into consideration all the percussion marks on a single bone element. We repeated this for the 10 bones in each series.
- Clusters taking into consideration one type of percussion mark (notch, pit and adhering flakes) for the whole series.

**Visual representation of clusters/mapping clusters.** To visually represent clusters, we followed Parkinson and colleagues’ method [18–20] for the Kernel density tool and implanted a new method using the Optimized Hot Spot Analysis tool. The Kernel Density tool counts the number of modifications in an area. This tool uses the quadratic kernel formula described in Silverman [39] to fit a smoothly tapered surface to each point, which spreads out to a specified radius around the point. The highest cell value is at the point located in the centre of the spread, with the value tapering to zero at the boundary of the search radius distance. The sum of the intersecting spreads is then calculated for each cell in the output raster [6]. Kernel Density analysis is to some extent subjective as the analyst must input a search radius and cell size, which can significantly influence the results. The analysis can be carried out with only two points and still display a cluster zone.

On the other hand, Optimized Hot Spot analysis is more objective and uses the Getis-Ord Gi* statistic to question the probability that a spatial distribution of values is random. To do this, the Optimized Hot Spot Analysis tool uses the average and median nearest neighbour calculations for aggregation and also to identify an appropriate scale of analysis. The tool computes each feature's average nearest neighbour distance and evaluates the distribution of all these distances. It defines high concentration zones (hot spots) and features that are more than a three-standard deviation distance away from their closest noncoincident neighbour, considered as locational outliers (cold spots). The limit of this tool, and the reason why we chose Kernel density for some analyses, is that Hot Spot analysis requires a minimum of 30 points to perform calculations. The numbers of observed percussion marks in our assemblage are not sufficient for testing all of our hypotheses with this tool.

Estimated cluster zones with the NND tool for separate bone elements are represented with Kernel density, for which we used a fixed search radius (0.12) and cell size (between 1.1 and 3.2). Analyses were not performed for areas with three impacts or less. Optimized Hot Spot analysis was used to estimate and illustrate clusters of all the percussion marks combined from the ten bones in a series.

We used Spearman’s *rho* to test for correlations between several values. The coefficients were calculated, and their statistical significance was tested using PAST software [40].

## Results

### Experimental bone breakage remains

The breakage of the 40 cattle long bones resulted in 1 219 fragments, for which element and side were identified in 549 cases (44%) (Table 4). 701 fragments (56%) could not be identified, however, in terms of weight, the unidentified fragments represent only 1% of the total assemblage. The tibia series generated the highest rate of fragments (number of specimens produced: NSP), while the femur series produced the lowest, but these results are reversed in terms of number of identified and refitted specimens (NISP%). Among the NISP, 288 fragments (Table 4) bear at least one percussion mark, whereas among the unidentified fragments 71 have percussion marks. These latter are not included in our study.

**Table 4:** Presentation of the studied faunal series and number of percussion marks.

*Bone Element*	*NSPª*	*NSP %*	*NISP^b^*	*NISP %*	*IND^c^*	*IND %*	*NISP with ^d^PM*	*NISP with PM %*	*IND* *with PM*	*IND with PM %*	*NISP weight (g)*	*NISP weight (g) %*	*IND weight (g)*	*IND weight (g) %*
***Humerus***	289	**24%**	108	**37%**	181	**63%**	66	**81%**	15	**19%**	11156	**98%**	179	**2%**
***Radius-ulna***	263	**22%**	119	**45%**	144	**55%**	61	**79%**	16	**21%**	8991	**97%**	108	**3%**
***Femur***	228	**19%**	130	**51%**	129	**49%**	68	**80%**	17	**20%**	18467	**99%**	98	**1%**
***Tibia***	439	**36%**	192	**44%**	247	**56%**	93	**80%**	23	**20%**	11207	**98%**	216	**2%**
***Total***	1219	**100%**	549	**44%**	701	**56%**	288	**80%**	71	**20%**	49821	**99%**	601	**1%**

ªNSP (number of specimens produced) = total number of fragments for each type of bone element

^b^NISP (Number of identified and refitted specimens)

^c^IND (unidentified specimens)

^d^PM (percussion marks) = number of percussion marks identified on each type of bone element.

**Fig 3:**
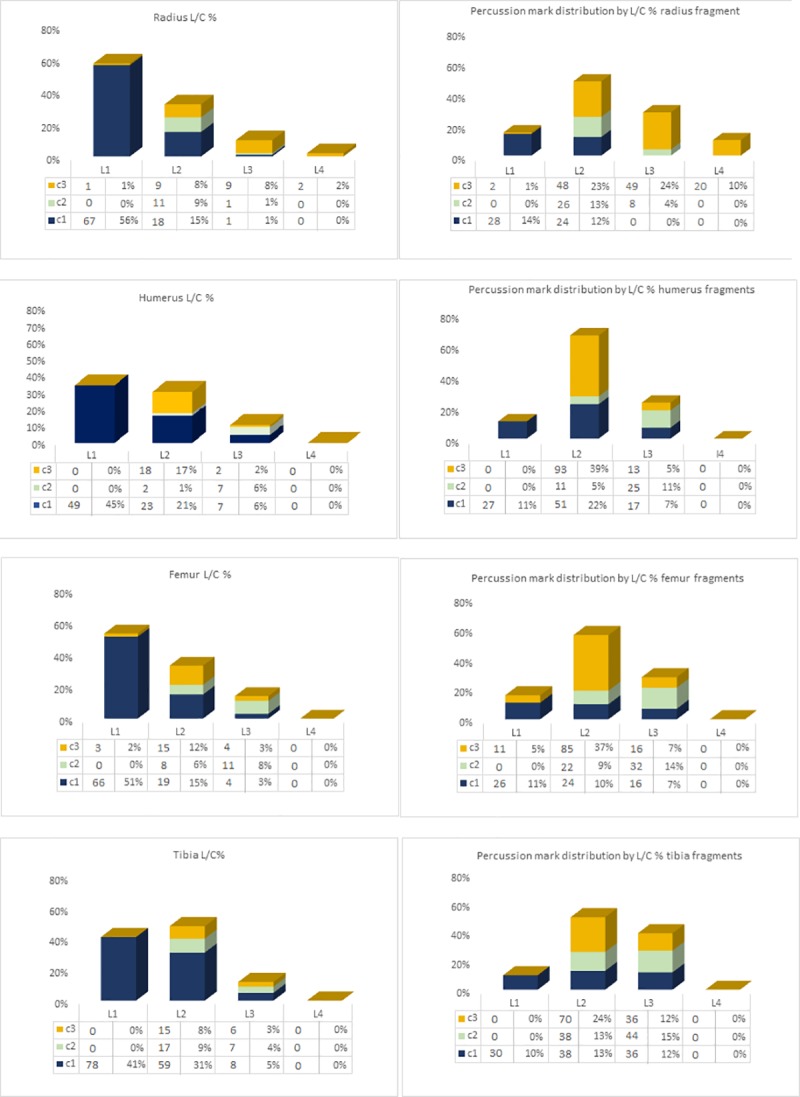
Distribution of NISP according to shaft length and circumference. Left column diagrams show relative frequencies of shaft length (L) and circumference (C) of all identified specimens; Right column diagrams show frequencies of percussion mark distribution according to relative frequencies of shaft length(L) and circumference (C) for specimens bearing percussion marks.

The majority of NISP have a L1 and L2 length (Fig 3). Fragments with L1 length almost all have a C1 circumference, while the L2 fragments display more diverse circumferences. Percussion marks are most frequently identified on fragments with an L2 length and a C3 circumference (Fig 3). Spearman’s test showed a positive correlation between the relative frequencies of shaft length and circumference for all identified specimens and the relative frequencies of percussion mark distribution of fragments bearing percussion marks (Humerus ρ = 0.09622 P = 0; Radius ρ = 0.85353 P = 0.00041, Femur ρ = 0.97268 P = 0 and Tibia ρ = 0 P = 0.00488.).

### Bone survivorship

**Fig 4:**
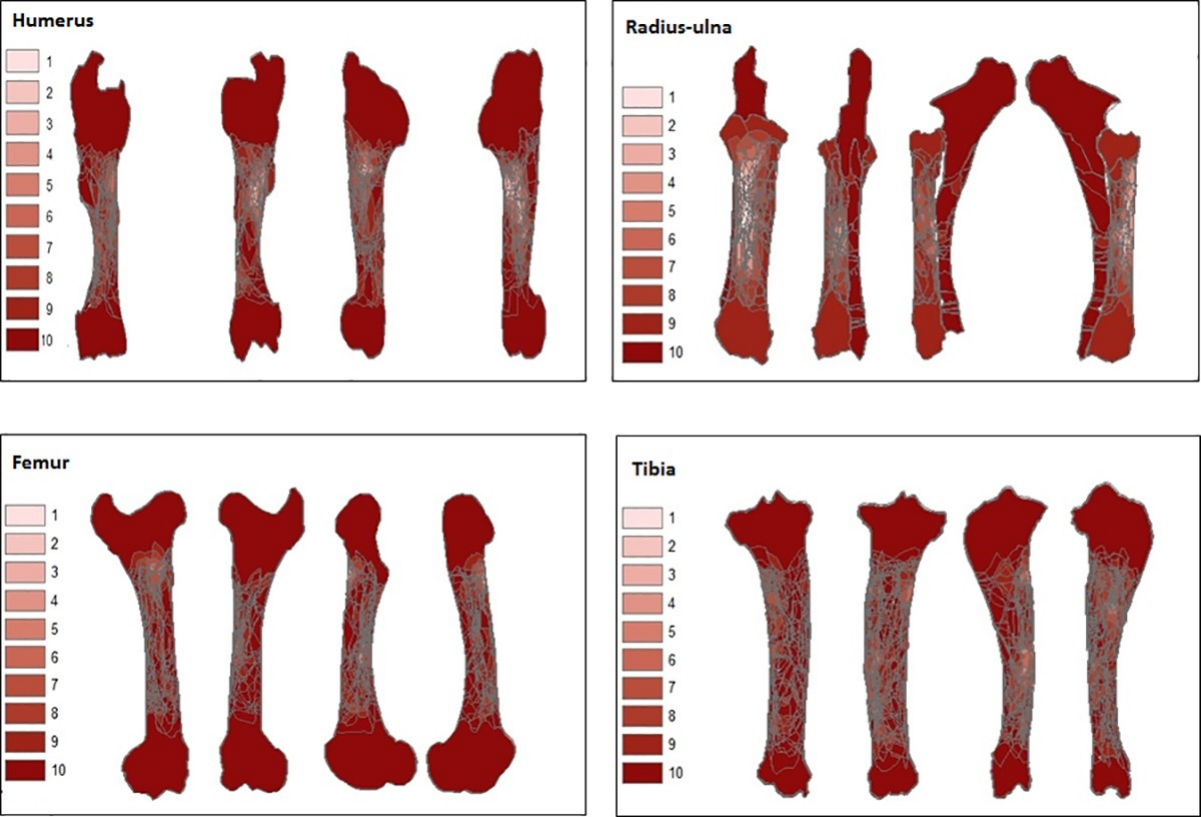
Bone survivorship composite of ten bone elements in a series for each bone element: Humerus, radius-ulna, femur and tibia represented in each side in the following order: Anterior, posterior, medial and lateral. Cortical preservation is indicated by shades of the colour red accompanied by a number from 1–10, darker shades and high numbers represent high cortical preservation.

Results of cortical bone survivorship for each series of the four elements are represented in the form of a graduated colour map generated with GIS (Fig 4), accompanied by the rate of preservation (Tables 5–8). The maps represent composites of both left and right elements in the assemblage. The darker shaded areas indicate higher cortical survivorship. The results show that destruction resulting from percussion for marrow extraction affects exclusively the diaphysis of long bones, but the zones concerned and the extent of destruction vary for the different elements.

**Table 5:** Series 1 humerus.

*Humerus ref. n°*	*Lateralisation*	*NISP^a^*	*Cortical preservation %^b^*	*Total n° of blows^c^*	*Number of PM per side*				*Total n° of PM^d^*	*PM frequency^e^*
					*anterior*	*posterior*	*medial*	*lateral*		
*1–1*	left	4	91%	110	2	1	4	11	18	20%
*1–2*	left	11	92%	78	3	0	7	10	20	22%
*1–2*	left	12	94%	93	0	8	9	2	19	20%
*1–4*	left	8	95%	38	0	2	8	9	19	20%
*1–5*	left	13	91%	40	3	4	4	12	23	25%
*1–6*	left	10	93%	50	13	4	9	4	30	32%
*1–7*	right	11	93%	51	11	1	3	3	18	19%
*1–8*	left	13	86%	50	4	3	8	11	26	30%
*1–9*	right	9	96%	32	8	4	7	4	23	24%
*1–10*	right	17	91%	30	9	15	15	2	41	45%
***Mean***		10.8	92%	57.2	5.3	4.2	7.4	6.8	23.7	26%
***Standard deviation***		3.4	2.8	27.2	4.6	4.4	3.4	4.1	7.2	8.1

^a^Number of identified specimens (NISP)

^b^Cortical preservation rate per element

^c^Total number of blows inflicted per element

^d^Total number of percussion marks (PM) per element and side

^e^PM frequency calculated according to cortical preservation.

**Table 6:** Series 2 radius.

*Radius ref. n°*	*Lateralisation*	*NISP^a^*	*Cortical preservation %^b^*	*Total n° of blows^c^*	*Number of PM per side*				*Total n° of PM^d^*	*PM frequency^e^*
					*anterior*	*posterior*	*medial*	*lateral*		
*2–1*	right	12	82%	115	12	3	6	4	23	30%
*2–2*	right	13	88%	88	18	8	0	3	28	33%
*2–3*	left	15	86%	48	8	12	0	0	19	23%
*2–4*	right	6	81%	94	10	3	0	3	14	20%
*2–5*	left	15	92%	29	16	2	0	0	17	20%
*2–6*	left	15	86%	108	13	11	0	5	27	34%
*2–7*	left	15	82%	131	14	9	0	3	25	32%
*2–8*	right	6	84%	71	6	4	1	6	16	20%
*2–9*	right	10	91%	67	17	4	0	2	22	25%
*2–10*	right	12	91%	53	9	6	0	1	15	17%
***Mean***		11.9	86%	80.4	12.3	6.2	0.7	2.7	20.6	25%
***Standard deviation***		3.5	4.1	32	4	3.6	1.9	2	5.2	6.3

^a^Number of identified specimens (NISP)

^b^Cortical preservation rate per element

^c^Total number of blows inflicted per element

^d^Total number of percussion marks (PM) per element and side

^e^PM frequency calculated according to cortical preservation.

**Table 7:** Series 3 femur.

*Femur ref. n°*	*Lateralisation*	*NISP^a^*	*Cortical preservation %^b^*	*Total n° of blows^c^*	*Number of PM per side*				*Total n° of PM^d^*	*PM frequency^e^*
					*anterior*	*posterior*	*medial*	*lateral*		
*3–1*	left	14	94%	83	11	2	4	5	21	23%
*3–2*	left	11	98%	56	3	5	6	1	14	15%
*3–3*	left	11	94%	87	8	1	9	12	29	32%
*3–4*	right	8	95%	41	13	2	2	10	26	18%
*3–5*	left	11	95%	55	10	3	7	11	30	33%
*3–6*	right	23	95%	30	11	2	13	1	26	28%
*3–7*	right	9	97%	50	11	3	2	2	17	19%
*3–8*	right	11	97%	12	1	4	12	6	22	24%
*3–9*	right	17	94%	49	16	6	4	8	33	36%
*3–10*	right	15	93%	37	8	5	0	2	14	16%
***Mean***		13	95%	50	9.2	3.3	5.9	5.8	23.2	24%
***Standard deviation***		4.4	1.6	22.6	4.5	1.6	4.3	4.3	6.7	7.5

^a^Number of identified specimens (NISP)

^b^Cortical preservation rate per element

^c^Total number of blows inflicted per element

^d^Total number of percussion marks (PM) per element and side

^e^PM frequency calculated according to cortical preservation.

**Table 8:** Series 4 tibia.

*Tibia ref. n°*	*Lateralisation*	*NISP^a^*	*Cortical preservation %^b^*	*Total n° of blows^c^*	*Number of PM per side*				*Total n° of PM^d^*	*PM frequency^e^*
					*anterior*	*posterior*	*medial*	*lateral*		
*–1*	left	22	92%	16	6	7	9	4	26	28%
*4–2*	left	19	96%	13	1	8	3	6	18	19%
*4–3*	left	16	92%	27	7	15	0	4	26	28%
*4–4*	left	20	98%	15	3	8	5	10	26	26%
*4–5*	left	21	90%	26	7	8	2	6	23	25%
*4–6*	right	22	93%	26	21	16	6	4	47	50%
*4–7*	right	24	90%	28	22	3	5	2	32	35%
*4–8*	left	14	94%	26	4	6	7	6	23	24%
*4–9*	right	18	95%	21	11	12	6	6	35	37%
*4–10*	right	16	95%	21	14	13	4	5	36	38%
***Mean***		19.2	94%	21.9	9.6	9.6	4.7	5.3	29.2	31%
***Standard deviation***		3.2	2.6	5.5	7.2	4.2	2.6	2.1	8.4	9

^a^Number of identified specimens (NISP)

^b^Cortical preservation rate per element

^c^Total number of blows inflicted per element

^d^Total number of percussion marks (PM) per element and side

^e^PM frequency calculated according to cortical preservation.

Highest cortical survivorship was calculated for the ulna series with an average of 99%. This is due to the nearly absent medullar cavity and scarcity of bone marrow in the element. Due to this constant high preservation and the low number of observed percussion marks, the ulna series was excluded from further analysis. Average cortical preservation for the rest of the elements is as follows: hindlimb bones show a preservation rate of 95% for the femur and 94% for the tibia series. For the front limbs, the humerus series presents an average of 92%, while the radius series shows an average of 86%. The most recurrently affected zone for all elements is the proximal to central part of the diaphysis, but this is not systematic for all sides. For the humerus and tibia series, zones of low preservation are rare on the anterior side. The femur posterior side shows no zones with low preservation. The radius series shows zones of low cortical preservation on all four sides.

### Patterns and cluster distributions

**Fig 5:**
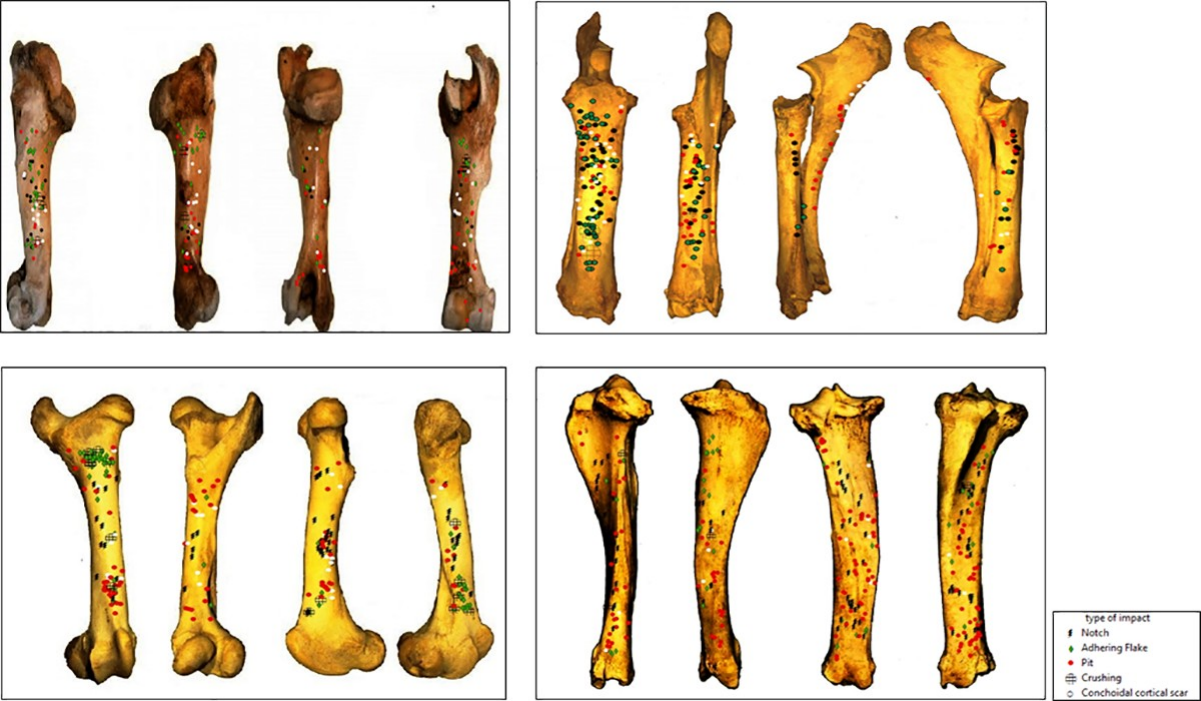
Distribution of percussion marks along the long bone elements (humerus, radius-ulna, tibia and femur), divided by type of percussion mark (each percussion mark is indicated with a specific symbol).

A total of 967 percussion marks were identified, comprising 237 in the humerus series, 206 in the radius, 232 in the femur and 292 in the tibia series. The most recurrent percussion marks in our sample are notches, pits andhering flakes (Table 9). Fig 5 shows obvious concentration zones for some percussion marks, e.g., the proximal part of the femur’s anterior side shows an important cluster of adhering flakes. However, it is more difficult to identify other marks that seem more dispersed or mixed up with others.

**Table 9:** Distribution of percussion marks by type of mark and element aspect.

*Element* *aspect*	*Type of percussion mark*	*Humerus*	*Radius*	*Femur*	*Tibia*	*Total*
***Anterior***	Notch	7 *(13%)*	32 *(29%)*	11 *(12%)*	34 *(35%)*	84 *(24%)*
	Pit	18 *(34%)*	16 *(14%)*	32 *(35%)*	45 *(47%)*	111 *(31%)*
	^a^Adh. Flake	13 *(25%)*	39 *(35%)*	34 *(37%)*	14 *(15%)*	100 *(28%)*
	^b^Crushing	4 *(8%)*	6 *(5%)*	8 *(9%)*	1 *(1%)*	19 *(5%)*
	^c^C. c. scar	11 *(21%)*	19 *(17%)*	7 *(8%)*	2 *(2%)*	39 *(11%)*
***Posterior***	Notch	7 *(17%)*	17 *(28%)*	5 *(15%)*	31 *(32%)*	60 *(26%)*
	Pit	18 *(43%)*	18 *(30%)*	18 *(55%)*	51 *(53%)*	105 *(45%)*
	Adh. Flake	10 *(24%)*	14 *(23%)*	3 *(9%)*	9 *(9%)*	36 *(16%)*
	Crushing	0	6 *(10%)*	0	0	6 *(3%)*
	C. c. scar	7 *(17%)*	5 *(8%)*	7 *(21%)*	5 *(5%)*	24 *(10%)*
***Medial***	Notch	9 (*12%)*	5 *(71%)*	21 *(36%)*	10 *(21%)*	45 *(24%)*
	Pit	28 *(38%)*	1 *(14%)*	22 *(37%)*	19 *(40%)*	70 *(37%)*
	Adh. Flake	19 *(26%)*	1 *(14%)*	5 *(8%)*	14 *(30%)*	39 *(21%)*
	Crushing	3 *(4%)*	0	2 *(3%)*	1 *(2%)*	6 *(3%)*
	C. c. scar	15 *(20%)*	0	9 *(15%)*	3 *(6%)*	27 *(14%)*
***Lateral***	Notch	20 *(29%)*	11 *(41%)*	11 *(23%)*	18 *(34%)*	60 *(31%)*
	Pit	11 *(16%)*	9 *(33%)*	11 *(23%)*	25 (*47%)*	56 *(29%)*
	Adh. Flake	24 *(35%)*	4 *(15%)*	18 *(38%)*	6 *(11%)*	52 *(27%)*
	Crushing	3 *(4%)*	0	5 *(10%)*	2 *(4%)*	10 *(5%)*
	C. c. scar	10 *(15%)*	3 *(11%)*	3 *(6%)*	2 *(4%)*	18 *(9%)*

Abbreviations

^a^Adh. Flake = Adhering flakes

^b^ Crush = Crushing mark

^c^C.C. scar = Cortical conchoidal scar

We compared the number of inflicted blows and percussion mark frequency to see if there is a correlation between the two values (Tables 5–8). None of the elements showed statistically significant correlation for the two variables (Humerus ρ = 0.56618 and P = 0.08797; Radius-Ulna ρ = 0.61968, P = 0.05602; Femur ρ = 0.06667 P = 0.85481 and Tibia ρ = 0.29632 P = 0.40578).

**Fig 6:**
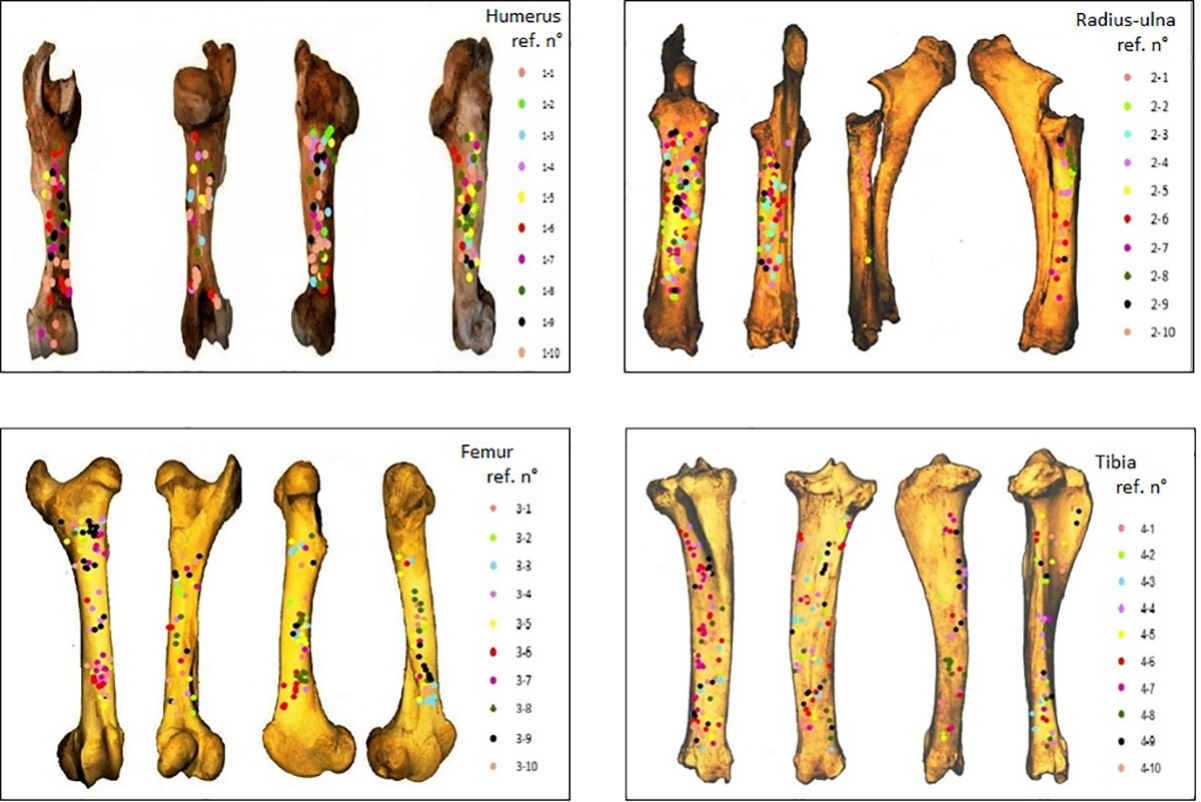
Distribution of percussion marks along the long bone elements, with different colours representing the marks made by different experimental attempts.

We also wanted to determine whether the experimenter found a more effective technique for breaking the bone over time and after several bone element breakages. For this, we ran a Spearman’s correlation test between the total number of inflicted blows and the number of experimental attempts (from 1 to 10) (Tables 5–8) (Fig 6). Results showed a significant negative correlation for the humerus (ρ = -0.77204 and P = 0.00888) and femur (ρ = -0.72121 and P = 0.01857), which means that the number of blows required to breach the cavity and fully extract the marrow tends to decrease from the first attempt to the last. Non-significant correlation was found for the radius (ρ = -0.22424 and P = 0.5334) and tibia series (ρ = 0.32004 and P = 0.36732).

#### Testing for clusters per element

Using the GIS average NND tool we determined the existence of cluster zones per side on each bone element (S1 Table). Results show percussion mark clusters on 37 out of 40 long bone elements. Only one radius element (ref n° 2–9) and two tibia elements (ref. n° 4–2 and 4–5) showed no significant clusters.

**Fig 7:**
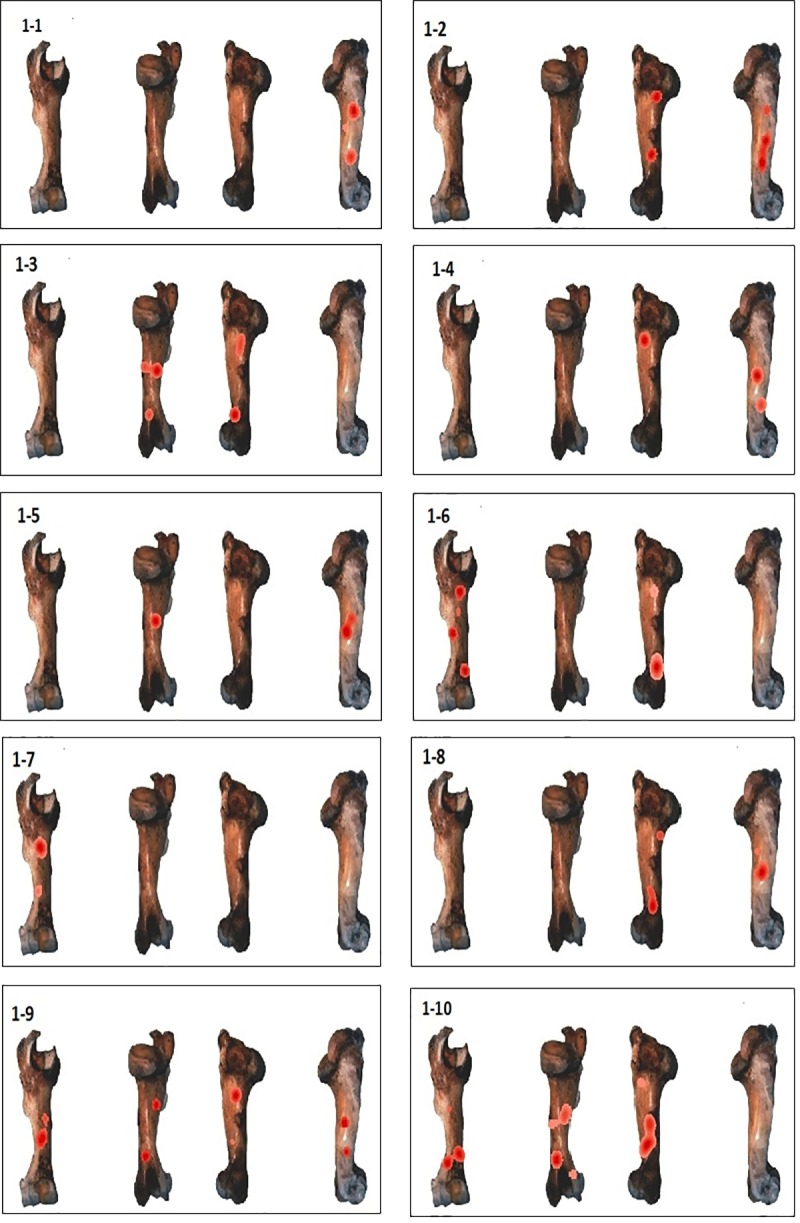
GIS Kernel density analysis results of percussion mark distribution on the four aspects for each humerus. Dark red areas indicate the highest concentrations of percussion marks.

**Fig 8:**
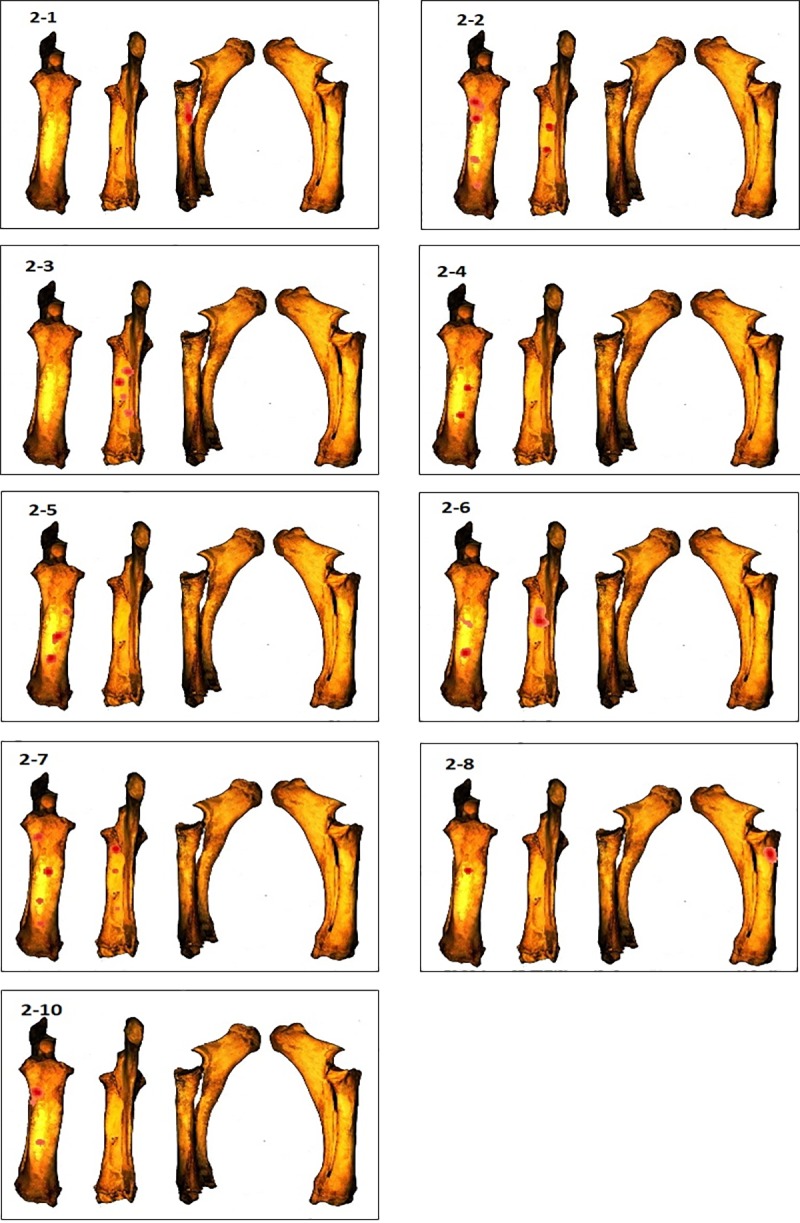
GIS Kernel density analysis results of percussion mark distribution on the four aspects for each radius. Dark red areas indicate the highest concentrations of percussion marks.

**Fig 9:**
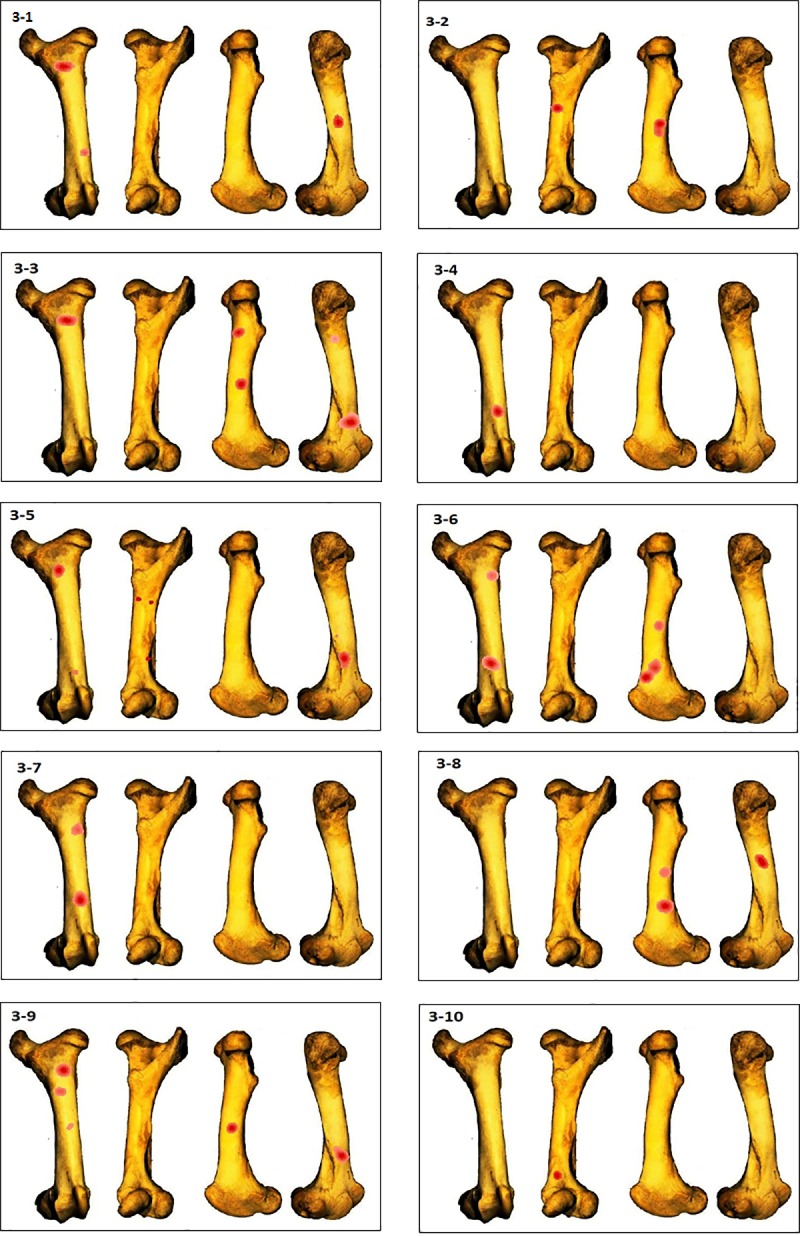
GIS Kernel density analysis results of percussion mark distribution on the four aspects for each femur. Dark red areas indicate the highest concentrations of percussion marks.

**Fig 10:**
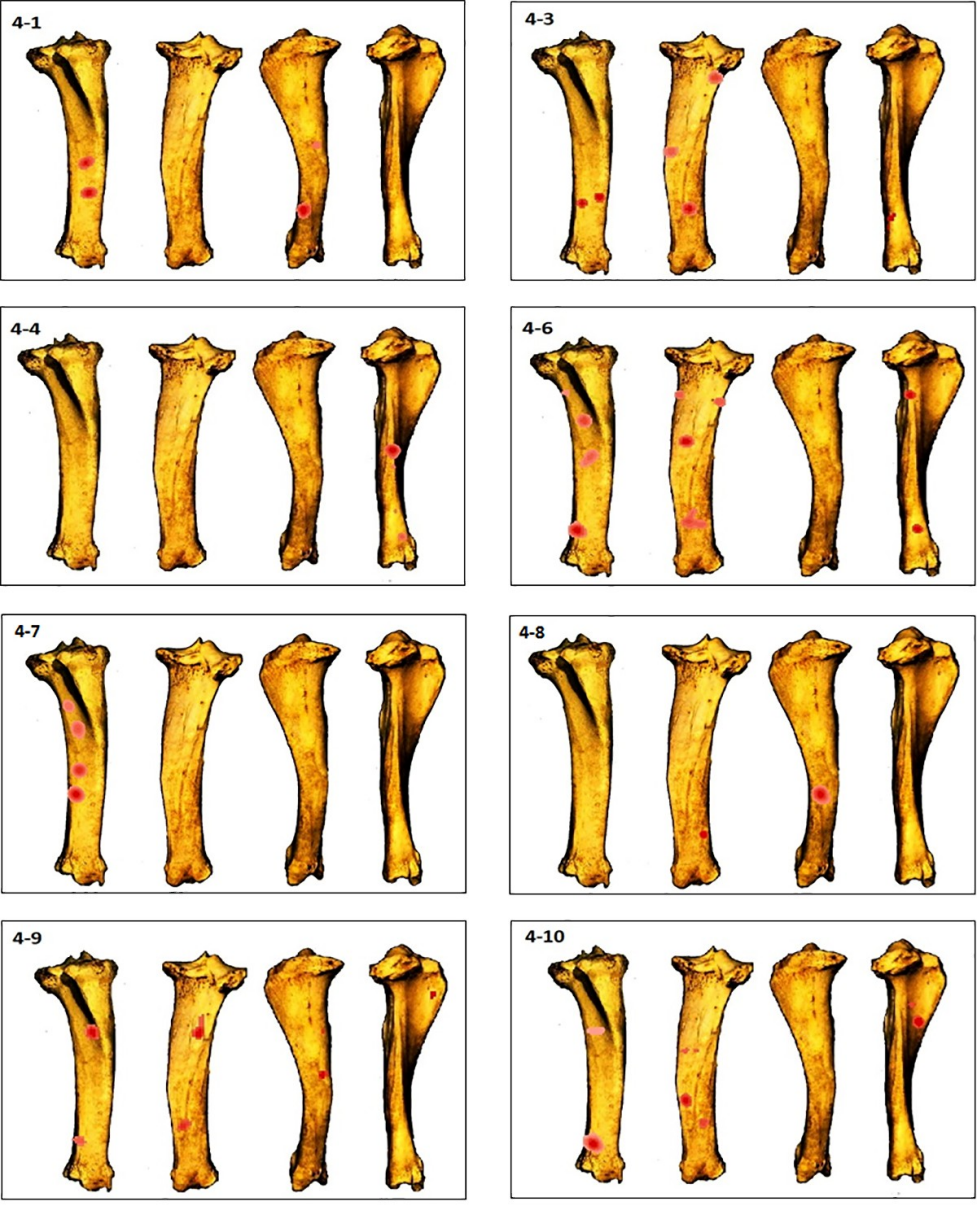
GIS Kernel density analysis results of percussion mark distribution on the four aspects for each tibia. Dark red areas indicate the highest concentrations of percussion marks.

**Fig 11:**
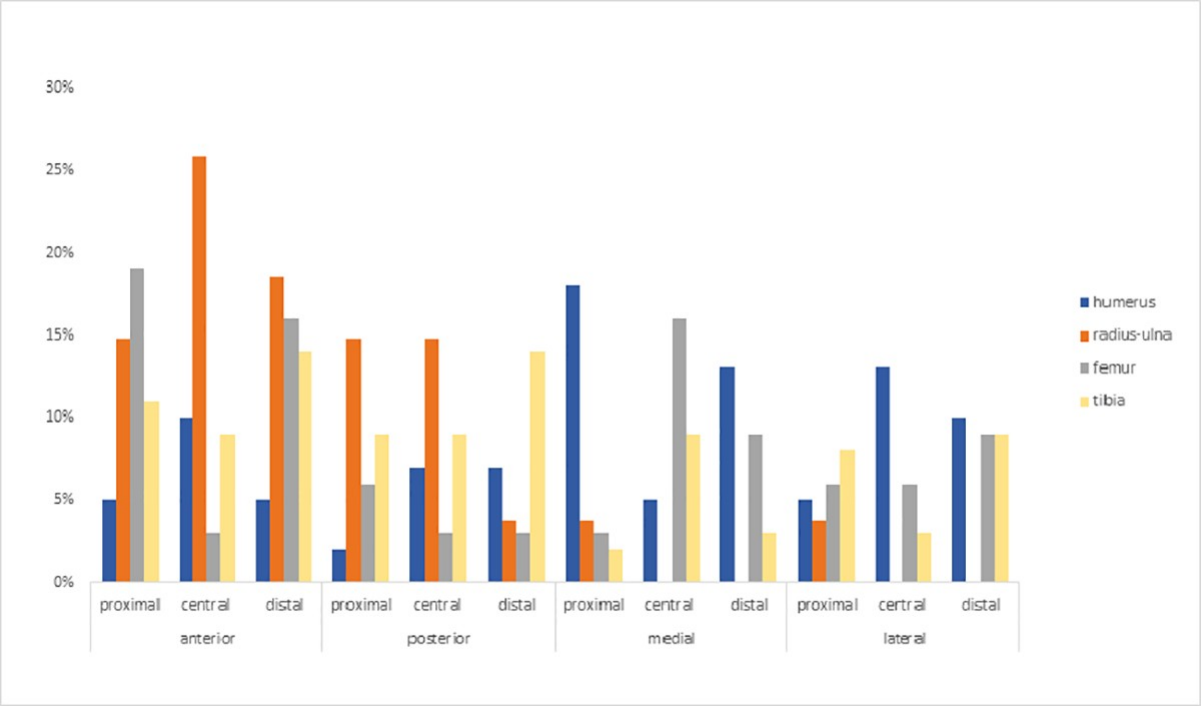
Summary of Kernel density cluster distribution according to side and shaft portion.

To illustrate the identified cluster zones, we used the kernel density tool (Figs 7–10), which shows significant variation in the distribution of these clusters across the different elements. The distribution of number of clusters by side (Anterior, Posterior, Medial and Lateral) and portion of the shaft (Proximal, Central, Distal) is calculated and summarized in Fig 11.

In the humerus series, clusters are most recurrent on the lateral and medial sides (Fig 7), (S1 Table). The most impacted zones on the lateral side are the central and distal parts of the diaphysis, and the proximal and distal parts of the diaphysis on the medial side. The identified clusters on the posterior and anterior sides are randomly distributed on the diaphysis (Fig 11). The radius series clusters are most frequently identified on the anterior side of the diaphysis (Fig 8), (S1 Table), with the highest rate on the central part (Fig 11). Posterior side clusters are less recurrent, but are consistent on the proximal and central parts of the diaphysis. The lateral and medial sides rarely show cluster zones, but the few representative zones are situated on the proximal part of the diaphysis. Femur clusters are most recurrent on the anterior and medial sides (Fig 9), (S1 Table). The anterior side clusters are located on the proximal and distal parts of the diaphysis, and the medial side clusters on the central and distal parts. Clusters on the posterior and lateral sides are less frequent and equally distributed on the shaft. Like the radius series, tibia clusters are most recurrent on the anterior and posterior sides, although with a lower rate. No single portion of the diaphysis can be differentiated (Fig 11).

**Fig 12:**
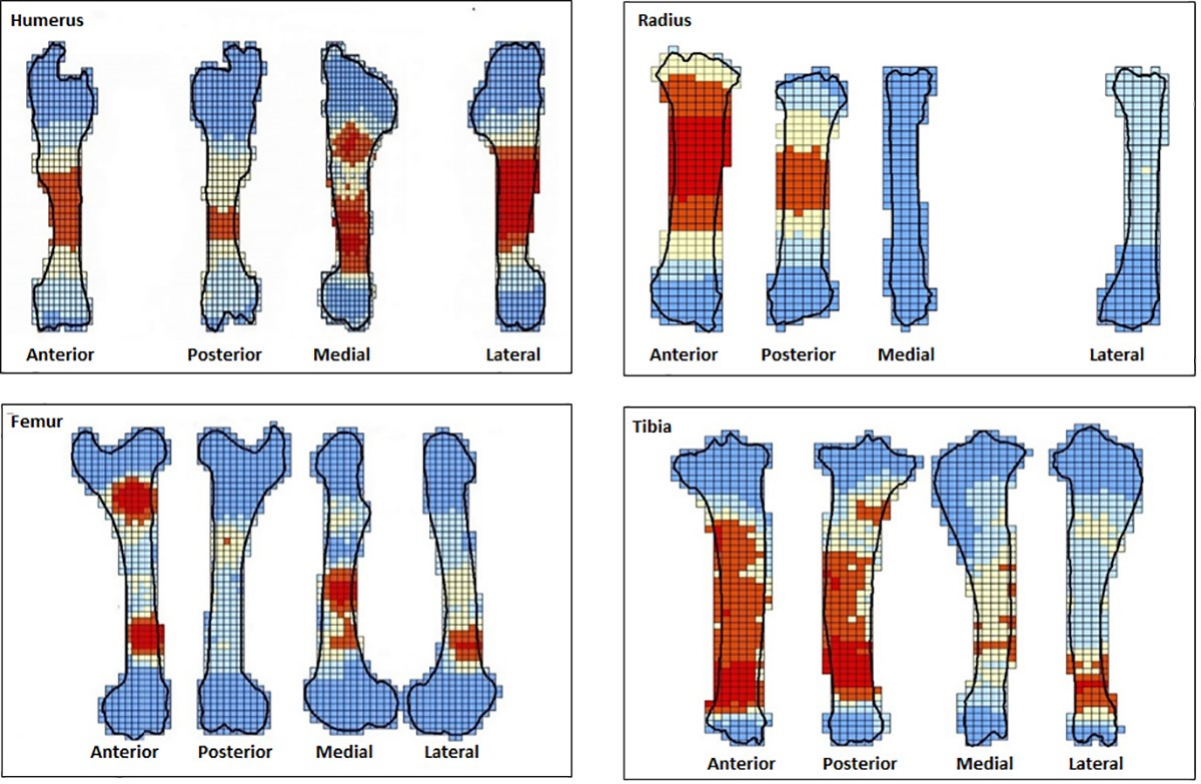
Optimized Hot spot analysis of combined percussion marks from the ten bone elements in each series.

Cluster patterns were identified for all the 10 bones of each series combined using Optimized hot spot analysis (Fig 12). The results represent a synthesis of the individual Kernel density map elements and reveal results corresponding to Fig 11. The advantage of Hot spot analysis is that it balances out the degree of clustering by taking into consideration the different views for the analysis.

In sum, the proximal and distal parts of the humerus diaphysis are cold spots, except for the medial side. The whole humerus diaphysis represents a hot spot zone for the lateral side. Anterior and posterior side hot spot zones display lower confidence levels of clustering (Fig 12) and correspond to the central to distal parts of the diaphysis.

The combined radius series percussion mark cluster analysis defines a high confidence hot spot zone on the anterior side of the diaphysis. The posterior side shows a well-defined hot spot zone on the proximal and central shaft. The lateral medial sides are identified as cold spots.

For the femur series, the well-defined hot spot zones are located on the anterior, proximal and distal parts of the diaphysis. Other significant hot spots are located on the medial central to distal part of the shaft and the distal part of the lateral shaft.

The most impacted sides for the tibia series are the anterior and posterior sides. However, the whole diaphysis is affected and the most impacted zone is the distal part of the shaft. The same zone is impacted for the lateral side as well.

#### Testing for clusters per type of percussion mark

**Fig 13:**
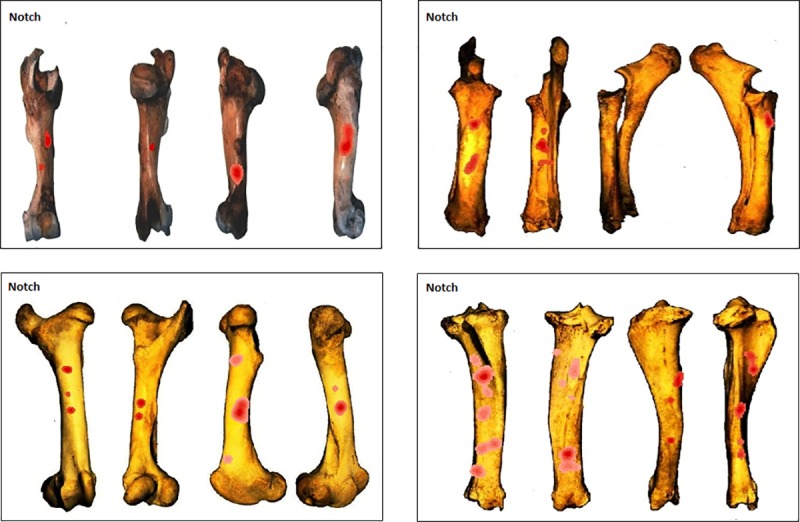
GIS Kernel density analysis results for notch distribution on the four aspects for each bone element series. Dark red areas indicate highest concentrations of notches.

**Fig 14:**
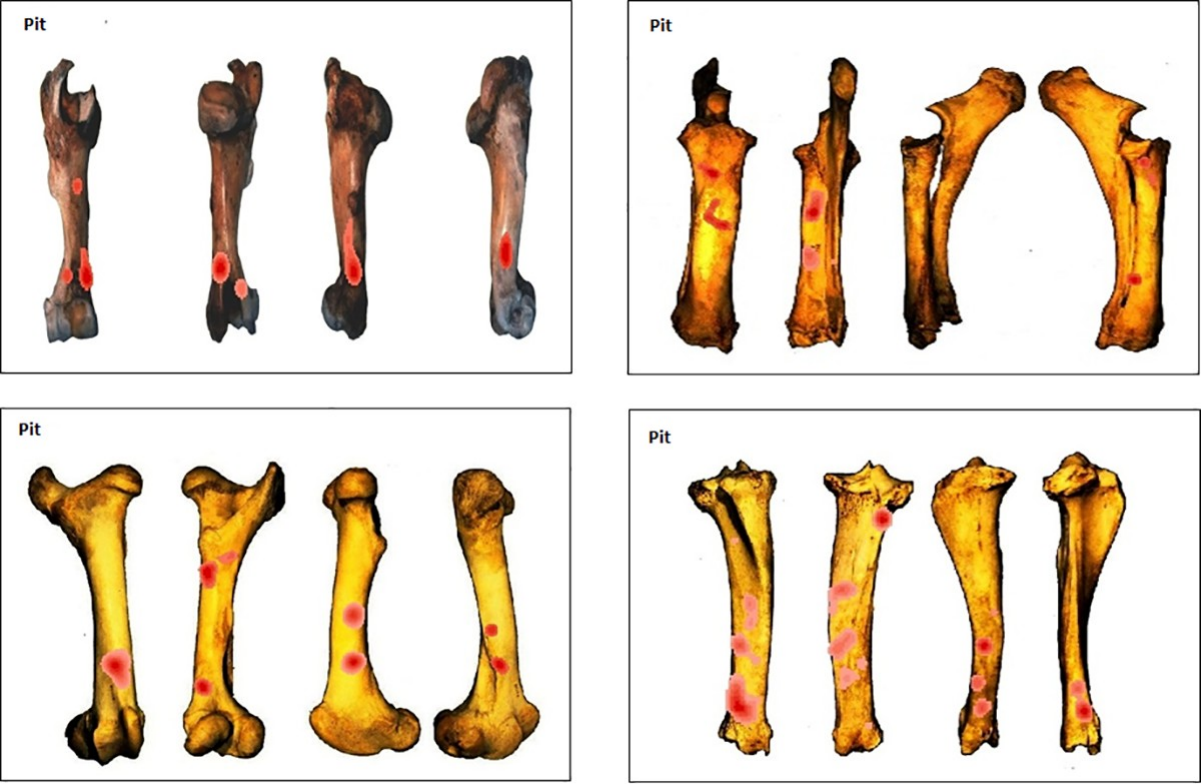
GIS Kernel density analysis results of pit distribution on the four aspects for each bone element series. Dark red areas indicate highest concentrations of pits.

**Fig 15:**
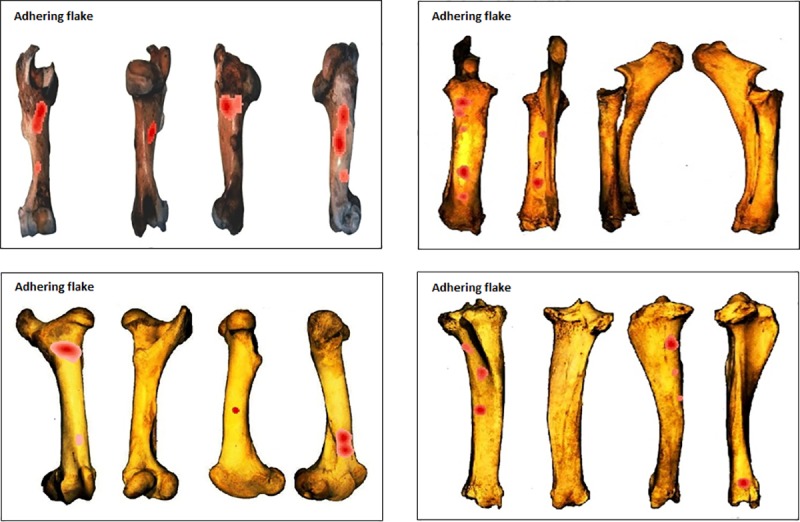
GIS Kernel density analysis results for adhering flake distribution on the four aspects for each bone element series. Dark red areas indicate highest concentrations of adhering flakes.

The three most recurrently identified percussion marks for the whole sample (notches, pits and adhering flakes) were tested using the NND tool (S2 Table) for clusters separately. Kernel density results show that while the overall patterns were quite distinct between the elements when all percussion marks were considered (Fig 12), similarities appear when we consider a single type of percussion mark. Notch clusters are more variable but frequently appear in the central part of the shaft (Fig 13). Pit clusters, with the exception of the radius series, are most recurrent on the distal part of the shaft (Fig 14). Adhering flake clusters are most recurrent in the proximal and distal parts of the shaft for all elements (Fig 15).

## Discussion

### Standardizing the documentation process

GIS software can be adapted and can provide valuable insights for non-geographical data, but it is important to clarify that this application has certain limitations. For the time being, georeferenced and uniformized templates constitute an appropriate option for establishing a workspace in GIS. However, this adjustment deforms all metric measurements, and therefore the drawings made using the software are not an exact replication. To adjust these values, most of the calculations can be expressed in frequency rates. Considering this, we underline the need for standardization and clear protocols for recording spatial data, i.e., precise criteria to define the placement of points (percussion marks) or outlines of polygons (fragments). If this is carried out correctly, the ArcGIS database can be used to organize and analyse big data and provide venues to explore a variety of questions in a short amount of time. For this study, we precisely calculated the examined surface and evaluated concentrations of percussion marks based on their spatial interdistances and not on imposed predefined portions. The principal advantage of GIS is the visual representation of the results. “Bone maps” of survivorship and clusters capture and convey information in a particularly insightful manner for the purpose of this study. This becomes clear when we compare figures with simply mapped features (Figs 5 and 6) to mapped clusters (Figs 7–12).

### Fragmentation and bone survivorship

Bone survivorship analysis evaluates bone loss, which is usually neglected in experiments. Here, all the fragments were directly identified as belonging to each element during the experiment and were collected, and in theory we should have approximately 100% bone survivorship, but this is not the case, due to several factors, referred to as laboratory taphonomy by Costamagno [41] and Bartram and Marean [42]. Costamagno discusses loss due to sampling procedures and analytical methods. In our case, part of the loss is, as mentioned before, due to the exclusive selection of the fragment’s cortical preserved surface. The range of fragment material loss is 5–14%. The weight of undetermined fragments does not exceed 3% in any element series and does not explain the low 86% average survivorship for the radius series. Other loss derives from putting the material through the different phases, starting from the experiment to the final step of the analysis. During breakage of the bone, one percent is lost immediately due to the crushing effect and the parting distance of small-sized fragments from the experiment spot. During the cleaning process, a certain number of fragments are lost or mixed up because of the large quantity of experimental material (400 bones), and cannot be reattributed to bones. The GIS method for bone survivorship proved to be effective because we not only calculate the percentage of the examined surface, but we can also visualize it and cross this data with percussion patterns and bone mineral properties. Crossing bone survivorship with clusters and patterns proved to be a better solution than the frequently used graphic representation of the relationship between the total recovered bone portions and bone portions with percussion marks. Indeed, it provides a visual representation of both and can reveal possible connections.

For calculating the fragmentation of our sample, we follow Marshall and Pilgram’s model of fragmentation effect [43]. As fragmentation intensity increases, the NISP decreases because fragments become so small as to be unidentifiable. The highest fragmentation rates in this sample were estimated for the tibia and humerus series, which showed high NSP but lower NISP rates (Table 4). However, this has no impact on cortical preservation, i.e., the examined surface did not decrease in size.

Pickering and Egeland’s hammerstone percussion experiment on deer humeri and radii elements, which also involved fracturing bones to a sufficient extent for complete marrow extraction, reports significantly higher fragmentation for the radii elements [28]. Moclan and Dominguez-Rodrigo [34] report similar results although the difference in fragmentation from their sample is less significant. Pickering and Egeland attribute this difference to the higher number of blows impacted on the radii to expose the medullary cavity (mean per individual radius = 6, humerus = 2). On the contrary, in our sample, the tibia series has the lowest number of impacted blows (mean per series = 21). In our experiment, the highest number of impacted blows was recorded for the radius series (mean per series: radius = 83), but this did not result in higher fragmentation, although it did result in the loss of 14% of the examinable surface, as shown by the bone survivorship results.

This difference can be explained by the variance in the concepts of the two experiments. The significant difference in the number of inflicted blows in these two experiments for the same bone element is due to the use of bones from different species. Indeed, deer bone elements are more gracile than cattle bones, and contrary to Pickering and Egeland’s experiment, volunteers in this study are novices or unexperienced in hammerstone percussion. Moreover, graduated bone survivorship “maps” (Figs 4–7) demonstrate that there is no constant fracture pattern. The numerous fragment outlines intersect, but rarely overlap, demonstrating multiple variable fracturing outcomes for the same bone element, even with standardization and systematization.

### Percussion mark clusters

Percussion marks were analysed per type of mark and per element with the NND and represented with the Kernel Density tool. The drawback with these tools is that illustrations and analyses are performed on a four-sided image of a bone element, which ignores the 3D shape of the bone. NND and Kernel density calculations are limited to one aspect of an element at a time, meaning that no connections can be made between the different aspects of the element (anterior, posterior, medial and lateral). This can be overcome with Optimized Hot Spot analysis. However, this tool requires a minimum of 30 features to conduct the analysis.

Cluster zones were examined in order to assess the degree of preservation of elements. Bone survivorship results show that zones with low cortical preservation (Figs 4–7) correspond well to clusters (hot spots) (Fig 12). Percussion marks themselves are read as negatives on the bone surface, i.e., zones that damage the cortical surface of the bone. In addition, when the experimenter inflicts additional blows to completely breach the shaft on a zone already bearing percussion marks, he/she obliterates them by crushing or reducing the size of the fragments to such an extent that they are no longer identifiable. In sum, the process of bone breakage itself reduces our ability to completely grasp marrow extraction by examining the material. Hence, during bone marrow extraction by humans, before sedimentary processes start acting, the most impacted zones by percussion are the least preserved zones, even in controlled conditions.

Abe et *al*. [16] state that “fragmentation generally decreases the number of cut marked fragments and cut mark counts relative to total fragments” (Abe et *al*. 2002:649 Abe et *al*. (2002:657) and that a “key assumption that all zooarchaeologists make is that more intensive cutting (more cutting actions) results in higher frequencies of cutmarks on the bone surface.” This is generally assumed for percussion marks as well. However, experiments by Egeland [44] found that there is a statistically significant negative correlation between the number of hammerstone blows and percussion mark frequency (r = -0.234, P = 0.045). In our sample, none of the elements showed statistically significant correlations for the two variables (Humerus ρ = 0.56618 and P = 0.08797; Radius-Ulna ρ = 0.61968 and P = 0.05602; Femur ρ = 0.06667 and P = 0.85481, Tibia *ρ = 0*.*29632 P = 0*.*40578*). We suspect that this is because the number of blows inflicted in Egeland is very low as experimenters had previous knowledge of hammerstone percussion. Several authors have underlined that professional butchers [14–30] or people practicing marrow extraction leave fewer traces on bone surfaces as their goal is to finish the task with the least possible effort. A person experimented in marrow extraction would be more efficient and would break the bone with fewer blows than a novice.

### Intuitive fracturing

We compared cortical bone survivorship and cluster analysis with Lyman’s density data [45] and Barba and Dominguez’s cortical thickness [46] data to evaluate whether the least preserved and most impacted surfaces can be correlated with fragile spots on the bone. GIS graduated maps of density and thickness measurements (S1 Fig) resulted in similar divisions and Spearman’s test showed a positive correlation for both values. Results showed that the least dense and least thick parts of the bone do not particularly correspond to low cortical preservation or cluster zones. The choice of impact points was not influenced by the bone’s mineral properties but possibly by the bones physical, morphological and physiological characteristics. These factors could result in unintentional patterns on the bone that may not be entirely guided by the individual’s intention but rather by the animal’s anatomy. For instance, the medial side of the humerus is flatter than the other sides, and thus more easily stabilized on the anvil. The video recording of the experiment showed that during the first blows to each humerus element, the volunteer recurrently put the medial side on the anvil and impacted the lateral side. In the final impacts, the individual flips the bone and impacts the medial side to completely break the shaft. That is why we see preferential sides when all percussion marks are combined (Fig 12). When we take into consideration a single type of percussion mark, the bone element’s morphology can also explain why preferential portions of the shaft were impacted. Notch clusters are most recurrent in the central portion of the shaft (Fig 13), where the hammerstone can strike the bone easily without slipping from the bone surface. Pit clusters appear on the distal part of the shaft (Fig 14), where the curved surface and epiphyses present obstacles for hammerstone impact. At this stage of the analysis, we cannot confirm or refute these statements, as every series was broken by a single individual. The analysis of the rest of the experimental material will help to check morphological conditioning factors.

## Conclusions

Through the study of this experimental sample, we observed the effectiveness of the GIS-based method and drew some conclusions relative to intuitive bone fracturing.

Bone morphology plays an important role in experimental bone breakage by beginners. Adaptation to the shape of the bone results in repetitive gestures and percussion mark pattern. These patterns are not perfectly standardized, but they show cluster zones of percussion marks for each bone element. Bone survivorship and a visual presentation of the examined surface show important links. Results in controlled experimental conditions show that the most impacted zones were the least preserved, and bone loss in the same zones would be much higher after the actions of taphonomic agents. Therefore, in archaeological assemblages, the connection between the examined surface and the pattern needs to be examined and clarified before making conclusions on the concentration and patterns of marks.

This study shows the potential of GIS spatial statistical abilities to improve existing quantifying methods and visually presenting the obtained results. The software provides a convenient system for stocking and analysing large data assemblages while considering the spatial link for input information. Analyses of modern experimental bone assemblages using the GIS method can provide valuable and easily shared analogues with which to compare percussion mark distributions in archaeological assemblages.

This study can also be used as a complementary experiment to Parkinson’s studies [18– 20] and assist in the determination of bone agent modifiers and their order of access, by adding hominid-induced marks to the large canid and felid database. It also underlines the importance of crossing bone survivorship with mark distribution, especially for archaeological assemblages, where taphonomic agents would considerably decrease the observable cortical surface. Crossing the two in a visual manner can relativize and provide information on the extent of fragmentation effects on percussion mark frequency.

## Supporting information

S1 Table GIS Cluster analysis for percussion marks for the four bone elements (humerus, radius, femur and tibia) using average nearest neighbour. Analysis was performed on the four aspects of the elements. The distance between each percussion mark and its nearest neighbour was measured. The nearest neighbour index (NNI) is the observed distance divided by the average expected distance in a hypothetical random distribution. If the NNI is less than 1, the pattern is considered clustered. If the NNI is greater than 1, the trend is towards dispersion. Colour codes (Z score) indicate the degree of clustering or dispersion. Null values signify that the number of counted marks was 3 or less.
(PDF)

S2 Table GIS Cluster analysis for individual type of percussion marks for the four bone elements (Humerus, Radius, Femur and Tibia) using average nearest neighbour. Analysis was performed on the four aspects of the elements. The distance between each percussion mark and its nearest neighbour was measured. The nearest neighbour index (NNI) is the observed distance divided by the average expected distance in a hypothetical random distribution. If the NNI is less than 1, the pattern is considered clustered. If the NNI is greater than 1, the trend is towards dispersion. Colour codes (Z score) indicate the degree of clustering or dispersion. Null values signify that the number of counted marks was 3 or less.
(PDF)

S1 Fig Graduated maps of thickness measurements according to Barba et al (2005). Darker blue zones represent the thicker portions of the bone.
(PDF)

S1 Protocol GIS percussion pattern analysis method. (PDF)
