# Supplementary material for: A GIS based approach to long bone breakage patterns derived from marrow extraction
Source: PLoS One. 2019 May 31;14(5):e0216733. doi: 10.1371/journal.pone.0216733 (PMC6544204; doi:10.1371/journal.pone.0216733)
Supplement: S1 Table — Analysis was performed on the four aspects of the elements. The distance between each percussion mark and its nearest neighbour was measured. The nearest neighbour index (NNI) is the observed distance divided by the average expected distance in a hypothetical random distribution. If the NNI is less than 1, the pattern is considered clustered. If the NNI is greater than 1, the trend is towards dispersion. Colour codes (Z score) indicate the degree of clustering or dispersion. Null values signify that the number of counted marks was 3 or less. (PDF) [file pone.0216733.s001.pdf]

Nearest neighbour distance (NND tool) results for all bone elements individually, taking all percussion marks into consideration :

Humerus												
ref. number	***Anterior***			***Posterior***			***Medial***			***Lateral***		
	p value	***z value***	***NNR***	***p value***	***z value***	***NNR***	***p value***	***z value***	***NNR***	***p value***	***z value***	***NNR***
**1-1**	Null	Null	Null	Null	Null	Null	0.154009	-1.425513	0.627428	0.000001	-4.83762	0.237562
**1-2**	Null	Null	Null	Null	Null	Null	0.000105	-3.878691	0.233689	0.000003	-4.64341	0.232451
**1-3**	Null	Null	Null	0.000077	-3.954895	0.269098	0.000051	-4.050458	0.294248	0.03454	-2.11371	0.362098
**1-4**	Null	Null	Null	0.000373	3.558783	2.315393	0.000296	-3.618388	0.331288	0.000082	-3.93735	0.313955
**1-5**	0.047816	-1.979002	0.402752	0.005525	-2.774719	0.274799	0.444417	-0.764755	0.821225	0.000215	-3.700529	0.441604
**1-6**	0.000027	-4.194251	0.414051	0.507612	0.66256	1.173167	0.000002	-4.708557	0.179581	0.192345	-1.30367	0.659272
**1-7**	0.028089	-2.196041	0.653891	Null	Null	Null	0.192554	-1.303062	0.606746	0.139798	-1.47654	0.554391
**1-8**	0.209394	-1.255233	0.671932	1.018086	0.05993	0.952212	0.007954	-2.654002	0.509515	0.014023	-2.45668	0.612812
**1-9**	0.001701	-3.13795	0.453244	0.000052	-4.047948	0.053721	0.002512	-3.021848	0.402975	0.00039	-3.54676	0.170881
**1-10**	0.000335	-3.586742	0.375046	0	-5.047863	0.318712	0	-5.201122	0.340611	0.091611	-1.68696	0.559096

Radius												
ref. number	***Anterior***			***Posterior***			***Medial***			***Lateral***		
	p value	***z value***	***NNR***	***p value***	***z value***	***NNR***	***p value***	***z value***	***NNR***	***p value***	***z value***	***NNR***
**2-1**	0.121284	-1.549407	0.7662	0.25536	-1.137428	0.734106	0.005643	-2.767832	0.409346	0.918138	-0.10278	0.973137
**2-2**	0.000003	-4.683532	0.422959	0.023538	-2.264585	0.605419	Null	Null	Null	Null	Null	Null
**2-3**	0.496279	-0.680357	0.874264	0.00016	-3.77445	0.43045	Null	Null	Null	Null	Null	Null
**2-4**	0.003471	-2.922646	0.516891	0.031424	2.151663	1.649356	Null	Null	Null	Null	Null	Null
**2-5**	0.034509	-2.114068	0.723734	0.174915	-1.356578	0.498583	Null	Null	Null	Null	Null	Null
**2-6**	0.000078	-3.94957	0.427405	0.000004	-4.58686	0.277083	Null	Null	Null	0.226308	-1.20992	0.717159
**2-7**	0.001209	-3.236683	0.547826	0.006305	-2.731473	0.524068	Null	Null	Null	0.000187	3.735329	2.127294
**2-8**	0.056768	-1.905096	0.056768	0.846399	-0.193715	0.954716	Null	Null	Null	0.000073	-3.96744	0.153349
**2-9**	0.346054	-0.942271	0.851492	0.189295	-1.312667	0.656921	Null	Null	Null	0	6.499282	3.402257
**2-10**	0.000064	-3.996332	0.303679	0.6839	-0.407147	0.913115	Null	Null	Null	Null	Null	Null

Femur												
ref. number	***Anterior***			***Posterior***			***Medial***			***Lateral***		
	p value	***z value***	***NNR***	***p value***	***z value***	***NNR***	***p value***	***z value***	***NNR***	***p value***	***z value***	***NNR***
**3-1**	0	-5.117315	0.19348	Null	Null	Null	0.113879	-1.580994	0.586791	0.001467	-3.181045	0.256375
**3-2**	Null	Null	Null	0.001372	-3.200572	0.25181	0.001085	-3.267545	0.302707	Null	Null	Null
**3-3**	0.018294	-2.359605	0.563923	Null	Null	Null	0.000003	-4.671422	0.186051	0	-5.63289	0.150018
**3-4**	0.000249	-3.663396	0.468894	Null	Null	Null	Null	Null	Null	Null	Null	Null
**3-5**	0.004996	-2.807313	0.535955	Null	Null	Null	0.081085	-1.744424	0.655355	0.000209	-3.707818	0.415626

**3-6**	0.000025	-4.212572	0.336073	Null	Null	Null	0	-5.315362	0.229398	Null	Null	Null
**3-7**	0.000026	-4.204824	0.337294	Null	Null	Null	Null	Null	Null	Null	Null	Null
**3-8**	Null	Null	Null	0.177242	-1.349296	0.647348	0.00023	-3.683239	0.444213	0.042587	-2.027735	0.567282
**3-9**	0.000828	-3.343286	0.563099	0.411488	-0.821277	0.82474	0.071829	-1.800203	0.529499	0.00001	-4.411089	0.184789
**3-10**	0.118583	-1.56075	0.711559	0.019745	-2.331159	0.455051	Null	Null	Null	Null	Null	Null

Tibia

ref. number	***Anterior***			***Posterior***			***Medial***			***Lateral***		
	p value	***z value***	***NNR***	***p value***	***z value***	***NNR***	***p value***	***z value***	***NNR***	***p value***	***z value***	***NNR***
**4-1**	0.000115	-3.856498	0.177025	0.135625	-1.492282	0.70517	0.037115	-2.084499	0.636797	0.120041	-1.554603	0.593689
**4-2**	Null	Null	Null	0.111567	-1.591191	0.705933	0.220718	1.224622	1.369582	0.113162	-1.584141	0.661945
**4-3**	0.028979	-2.183768	0.568554	0.006349	-2.729212	0.63165	Null	Null	Null	0.030797	-2.159689	0.435544
**4-4**	0.60734	-0.513874	0.844917	0.309281	-1.016731	0.812099	0.53727	-0.616947	0.855778	0.002107	-3.074724	0.491752
**4-5**	0.400276	-0.841129	0.833819	0.242597	-1.168521	0.784046	Null	Null	Null	0.099689	-1.646363	0.648667
**4-6**	0	-5.07555	0.421048	0.000153	-3.785597	0.505298	0.185561	1.323826	1.282504	0.001078	-3.269452	0.145496
**4-7**	0.000011	-4.404676	0.509124	0.000002	4.797897	2.447969	0.033033	2.131684	1.498319	Null	Null	Null
**4-8**	0.449612	-0.756062	0.802396	0.084441	-1.72548	0.631783	0.000028	-4.188252	0.172529	0.377044	-0.883356	0.811492
**4-9**	0.000314	-3.603695	0.432036	0.023538	-2.26459	0.658282	0.018291	-2.359681	0.496445	0.079796	-1.751871	0.626152
**4-10**	0.000069	-3.978926	0.444133	0.028162	-2.195028	0.681773	0.159294	-1.407449	0.632149	0.000972	-3.298565	0.228903

Dispersed 99% confidence
Dispersed 90-95% confidence
Random
Clustered 90-95% confidence
Clustered 99% confidence

S1 Table GIS Cluster Analysis for percussion marks for the four bone elements (Humerus, Radius, Femur and Tibia) using Average Nearest Neighbour. Analysis was performed on the four aspects of the elements. The distance between each percussion mark and its nearest neighbour was measured. The nearest neighbour index (NNI) is the observed distance divided by the average expected distance in a hypothetical random distribution. If the NNI is less than 1, the pattern is considered clustered. If the NNI is greater than 1, the trend is towards dispersion. Colour codes (Z score) indicate the degree of clustering or dispersion. Null values signify that the number of counted marks was 3 or less.
