# Supplementary material for: A GIS based approach to long bone breakage patterns derived from marrow extraction
Source: PLoS One. 2019 May 31;14(5):e0216733. doi: 10.1371/journal.pone.0216733 (PMC6544204; doi:10.1371/journal.pone.0216733)
Supplement: S2 Table — Analysis was performed on the four aspects of the elements. The distance between each percussion mark and its nearest neighbour was measured. The nearest neighbour index (NNI) is the observed distance divided by the average expected distance in a hypothetical random distribution. If the NNI is less than 1, the pattern is considered clustered. If the NNI is greater than 1, the trend is towards dispersion. Colour codes (Z score) indicate the degree of clustering or dispersion. Null values signify that the number of counted marks was 3 or less. (PDF) [file pone.0216733.s002.pdf]

Nearest neighbour distance (NND tool) results for ten bone elements in a series taking into consideration a single type of percussion mark:

Humerus												
ref. number 1 (1-10)	***anterior***			***posterior***			***medial***			***lateral***		
	**p value**	**z value**	**NNR**	**p value**	**z value**	**NNR**	**p value**	**z value**	**NNR**	**p value**	**z value**	**NNR**
notch	0.02612	-2.2244	0.5889	0.005007	-2.8066	0.26647	0.051413	-1.9480	0.66058	0	-5.0347	0.39623
pits	0.000002	-4.7462	0.4152	0	-5.1272	0.30800	0	-5.9376	0.35283	0.000047	-4.0692	0.29098
adhering flakes	0.000064	-3.9985	0.4204	0.03628	-2.0938	0.63518	0.000411	-3.5332	0.56469	0	-6.3233	0.35177
Radius												
ref. number 2 (1-10)	***anterior***			***posterior***			***medial***			***lateral***		
	**p value**	**z value**	**NNR**	**p value**	**z value**	**NNR**	**p value**	**z value**	**NNR**	**p value**	**z value**	**NNR**
notch	0.002293	-3.049355	0.718225	0.000033	-4.153019	0.473488	0.739162	-0.332963	0.928946	0.003008	-2.96692	0.532396
pits	0.005216	-2.793406	0.645857	0.000038	-4.119638	0.550981	Null	Null	Null	0.04694	-1.986842	0.671578
adhering flakes	0	-5.66379	0.525928	0.004654	-2.830062	0.604632	Null	Null	Null	0.282578	1.074547	1.280843
Femur												
ref. number 3 (3-10)	***anterior***			***posterior***			***medial***			***lateral***		
	**p value**	**z value**	**NNR**	**p value**	**z value**	**NNR**	**p value**	**z value**	**NNR**	**p value**	**z value**	**NNR**
notch	0.001855	-3.112576	0.509439	0.009957	-2.577308	0.397509	0	-6.026614	0.312563	0.002212	-3.060236	0.517688
pits	0	-5.362333	0.504495	0.000208	-3.708988	0.543029	0	-5.116608	0.429783	0.000502	-3.479535	0.451604
adhering flakes	0	-6.452415	0.421568	Null	Null	Null	0.007663	-2.666581	0.37664	0	-6.140227	0.243485
Tibia												
ref. number 3 (4-10)	***anterior***			***posterior***			***medial***			***lateral***		
	**p value**	**z value**	**NNR**	**p value**	**z value**	**NNR**	**p value**	**z value**	**NNR**	**p value**	**z value**	**NNR**
notch	0	-6.674831	0.401630	0.000001	-4.860112	0.543717	0.00003	-4.17716	0.309521	0.00025	-3.662662	0.548737
pits	0	-6.697298	0.478130	0.000001	-4.99054	0.634715	0.000203	-3.715334	0.554456	0.000379	-3.554238	0.628426
adhering flakes	0.000859	-3.332895	0.534385	0.869513	0.164277	1.028624	0.000149	-3.793439	0.470046	0.015772	-2.414143	0.484823

Dispersed 99% confidence

Dispersed 90-95% confidence
Random
Clustered 90-95% confidence
Clustered 99% confidence

S2 Table GIS Cluster Analysis for individual type of percussion marks for the four bone elements (Humerus, Radius, Femur and Tibia) using Average Nearest Neighbour. Analysis was performed on the four aspects of the elements. The distance between each percussion mark and its nearest neighbour was measured. The nearest neighbour index (NNI) is the observed distance divided by the average expected distance in a hypothetical random distribution. If the NNI is less than 1, the pattern is considered clustered. If the NNI is greater than 1, the trend is towards dispersion. Colour codes (Z score) indicate the degree of clustering or dispersion. Null values signify that the number of counted marks was 3 or less.
