# Supplementary material for: A GIS based approach to long bone breakage patterns derived from marrow extraction
Source: PLoS One. 2019 May 31;14(5):e0216733. doi: 10.1371/journal.pone.0216733 (PMC6544204; doi:10.1371/journal.pone.0216733)
Supplement: S1 Fig — Darker blue zones represent the thicker portions of the bone. (PDF) [file pone.0216733.s003.pdf]

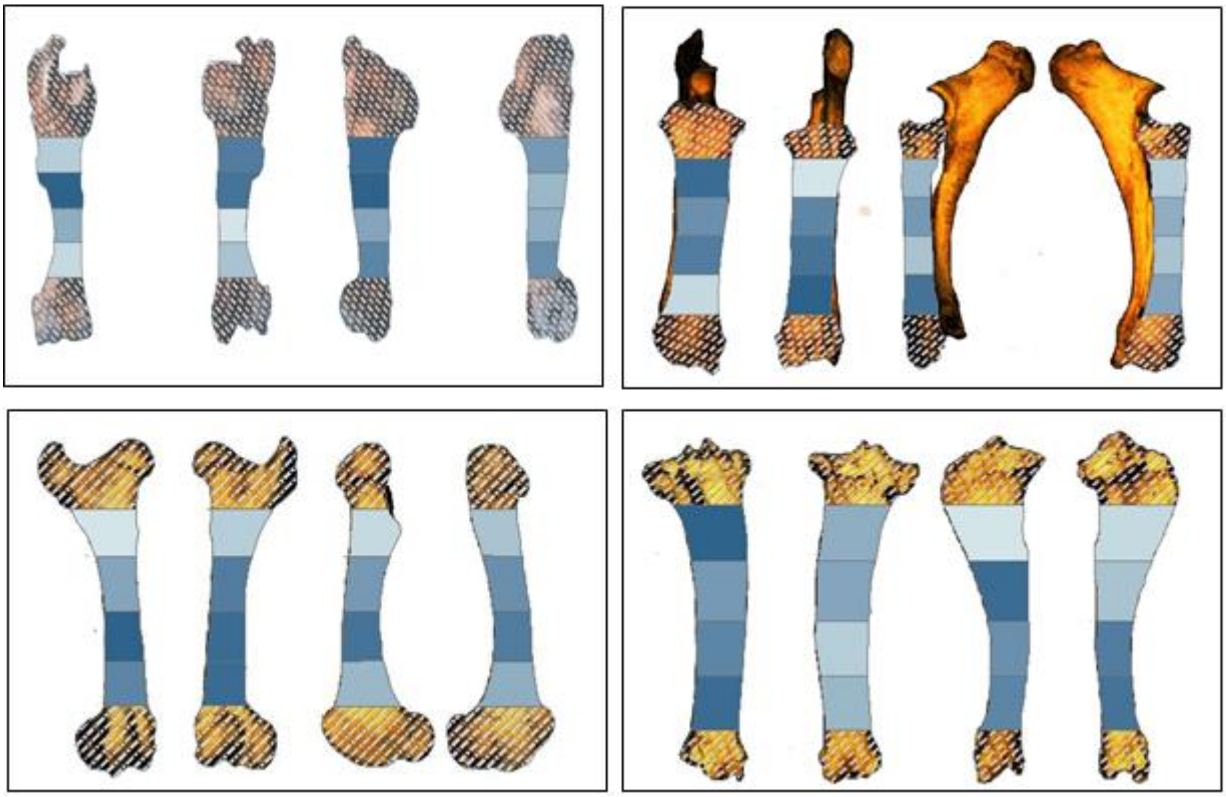

S1 Fig. Graduated maps of thickness measurements according to Barba et al. (2005) for humerus, radius, femur and tibia.

Darker blue zones represent the thicker portions of the bone.
