# Supplementary material for: A GIS based approach to long bone breakage patterns derived from marrow extraction
Source: PLoS One. 2019 May 31;14(5):e0216733. doi: 10.1371/journal.pone.0216733 (PMC6544204; doi:10.1371/journal.pone.0216733)
Supplement: S1 Protocol — (PDF) [file pone.0216733.s004.pdf]

GIS percussion pattern analysis method

The GIS method for analysing percussion mark patterns is available on the site protocols.io Follow the link to access the step by step described method: https://www.protocols.io/private/34F6E71E3177B8E0F106B2A9369DD2C2

S1 Protocol GIS percussion pattern analysis method
